# Prenatal exposure to nicotine and postpartum depression: a systematic review and meta-analysis

**DOI:** 10.1007/s00737-026-01739-6

**Published:** 2026-07-01

**Authors:** Reem Sharaf-Alddin, May Salama, Denise McKinney, George Saade, Qi Zhang

**Affiliations:** 1 Joint School of Public Health, Old Dominion University, Norfolk, United States; 2 Ellmer College of Health Sciences, Old Dominion University, Norfolk, United States; 3 Virginia Health Sciences, Old Dominion University, Norfolk, United States

**Keywords:** Smoking, Nicotine, Pregnancy, Postpartum depression, Meta-analysis

## Abstract

**Purpose:**

Postpartum Depression (PPD) is the most common mental health complication of childbirth, and has short-term and long-term consequences for mother, child, and the entire family. PPD is a function of several factors. Prenatal exposure to nicotine (PEN) by Active Tobacco Smoking (ATS), Secondhand Smoking (SHS), or Electronic Nicotine Products (ENPs) is a major potential risk factor. This systematic review and meta-analysis aim to summarize and evaluate all evidence regarding the association between PEN and PPD.

**Methods:**

We searched the following databases, PubMed, Medline, Cochrane, EMBASE, and CINHAL, for studies published between Jan.1st ,2000&Sep.19th ,2024, with the outcome PPD. Studies quality was assessed using the Newcastle–Ottawa Scale. Pooled Prevalence (PP) was calculated using the Freeman-Tukey Double Arcsine Transformation method. Pooled Odds Ratio (pooled-OR) was estimated using a random-effects model. Sensitivity analysis was conducted using the leave-one-out technique.

**Results:**

Of 29 studies in the systematic review, 26 included in the meta-analysis. PP of PPD was 0.15,(95% CI[0.12–0.17]; I^2^ = 99.80%,*p* < 0.001). Pooled-OR for ATS 1.96,(95%CI[1.59–2.41]; Z = 6.46,*p* < 0.001), 1.22,(95%CI[0.69–2.18], Z = 0.69,*p* = 0.49) for SHS, and 1.14,(95%CI[0.82–1.58,Z = 0.78,*p* = 0.43) for ENPs. While the overall Pooled-OR for all PEN was 1.74,(95%CI[1.44–2.12]; Z = 5.59,*p* < 0.001), funnel plots and Egger test showed no evidence of publication bias. Sensitivity analysis confirmed the robustness of the findings.

**Conclusion:**

This meta-analysis reveals that the accumulated evidence shows a significant association between prenatal PEN and PPD; though this final finding should be interpreted cautiously due to the high heterogeneity. Therefore, the exposure-stratified estimates represent the principal findings of this meta-analysis. The findings indicates a significant association between prenatal ATS and PPD, but no clear conclusion could be drawn about prenatal SHS or ENPs use due to the limited number of studies. There is a need for future studies that prospectively assess the impact of SHS and ENP on PPD.

**Supplementary Information:**

The online version contains supplementary material available at https://doi.org/10.1007/s00737-026-01739-6.

## Highlights

Pooled prevalence of postpartum depression indicates that screening tools overestimate the prevalence compared to diagnostic tools.

Prenatal Active tobacco smoking is associated with increased Odds of PPD.

No significant association was found between prenatal secondhand smoking and PPD.

**Supplementary Information:**

The online version contains supplementary material available at https://doi.org/10.1007/s00737-026-01739-6.

## Introduction

Postpartum depression (PPD) is a common and serious mood disorder that affects women within the first year after childbirth and can persist for several months. It is the most prevalent mental health condition in the postpartum period. (Wang et al. 2021a, b; Worthen and Beurel 2022) Globally, approximately 17% of women experience PPD, (Wang et al. 2021a, b) which varies widely across countries. PPD has both short- and long-term consequences for maternal health, (Darcy et al. 2011; Gagliardi et al. 2012; Kim et al. 2015; Lilja et al. 2012; Park et al. 2009; Pope et al. 2013) as well as significant impacts on child development, (Hay et al. 2003; “Maternal depression and child development,” 2004) and overall family well-being. (Letourneau et al. 2012; Paulson et al. 2006) It is associated with impaired child growth, (Avan et al. 2010; Farías-Antúnez et al. 2018; Holm-Larsen et al. 2019) delayed motor and language development, (Wang et al. 2024) and disrupted mother-infant emotional bonding. (Diaz-Ogallar et al. 2024) Women affected by PPD are more likely to have suicidal thoughts, (Yu et al. 2024) and are at increased risk of PPD recurrence in subsequent pregnancies. (Goodman 2004) Furthermore, maternal PPD is a strong predictor of paternal depression. (Thiel et al. 2020; D. Wang et al. 2021a, b)

Prenatal exposure to nicotine (PEN) including active tobacco smoking (ATS), secondhand smoke (SHS), and the use of electronic nicotine products (ENPs), has emerged as a potentially modifiable risk factor for PPD. Biological plausibility is supported through several interrelated pathways. Nicotine disrupts mood-regulating neurotransmitters like serotonin, dopamine, and norepinephrine, increasing susceptibility to depressive symptoms. (Quattrocki et al. 2000) It also activates the HPA axis, elevating cortisol and stress reactivity during and after pregnancy, both linked to PPD. (Steptoe and Ussher 2006) Nicotine also crosses the placenta and alters placental function, potentially disrupting maternal–fetal hormonal signaling, which may in turn affect maternal mood regulation. (Jauniaux and Burton 2007; Zdravkovic et al. 2005) Additionally, epigenetic changes resulting from nicotine exposure may affect gene expression related to emotional regulation and stress response, increasing susceptibility to depression. (Gould 2023) Moreover, nicotine’s psychoactive effects are clinically relevant, as it provides short-term mood regulation by increasing arousal, improving concentration and reward sensitivity, and temporarily alleviating stress and anxiety. (Benowitz 2009) However, abrupt cessation during pregnancy can trigger withdrawal symptoms, including irritability, low mood, anxiety, insomnia, and anhedonia, which mimic or exacerbate depressive states. (Hughes and Hatsukami 1986) These symptoms can reach levels comparable to those observed in psychiatric outpatients, (Hughes 2006) likely due to hedonic dysregulation and dopamine deficiency resulting from chronic nicotine exposure and withdrawal. (Koob and Le Moal 1997) Furthermore, psychosocial factors such as stigma, socioeconomic stress, and reduced access to healthcare, commonly associated with maternal smoking, may exacerbate PPD risk. (Gavin et al. 2005; Kahn et al. 2002; Tong et al. 2009)

Estimates of PEN vary widely across studies. Globally, the prevalence of prenatal ATS ranges from 1.8% to 32% (Jafari et al. 2021; Lange et al. 2018), prenatal SHS from 6% to 73% (Reece et al. 2019), and prenatal ENP use from 1.2% to 6.5%. (Liu et al. 2021; Obisesan et al. 2020; Rollins et al. 2020; Wagner et al. 2017) Although multiple studies have investigated the association between prenatal ATS or SHS and PPD, (Chen et al. 2019; Choi et al. 2024; Cui et al. 2020) findings remain inconclusive. To date, only three systematic reviews have examined this association. The first one focusing on ATS, which included 13 studies and was published in 2017. (Chen et al. 2019) The second review was focused on prenatal exposure to SHS and PPD. It included only three studies and was published in 2018. (Suzuki et al. 2019) The most recent one was published in 2025. This systematic review was confined only to prenatal ATS and limited to 4 years span. (Knysak et al. 2025) All of these previous reviews were limited to a single type of PEN (ATS or SHS), no previous review incorporated exposure to nicotine by Electronic Nicotine Products, and no previous review integrated the three types of PEN in provided pooled estimate of their association to PPD. To the best of our knowledge, no systematic review has evaluated prenatal ENP use, nor has any comprehensively assessed the overall impact of any form of PEN on PPD. This limits our understanding of nicotine’s broader implications. Therefore, this systematic review and meta-analysis aim to (1) summarize PPD prevalence among women exposed to all types of nicotine exposure during pregnancy and (2) estimate the pooled effect size of the association between PEN and PPD, informing public health strategies such as smoking cessation, exposure to SHS, and prenatal care interventions.

## Materials and methods

### Study design

This systematic review and meta-analysis are registered in PROSPERO (no CRD42024561898). The reporting quality was assessed in accordance with the Preferred Reporting Items for Systematic Reviews and Meta-Analysis (PRISMA) guidelines, and Population, Exposure, Comparison, Outcome, Time (PECOT) framework was used to define eligibility.

## PECOT

P: Pregnant women in all trimesters.

E: All forms of Exposure to Nicotine, such as Active Tobacco Smoking, Secondhand Smoking, and the use of Electronic Nicotine Products.

C: Women who were not exposed to/did not use nicotine during pregnancy (no Active Tobacco Smoking, no Secondhand Smoking, no use of Electronic Nicotine Products).

O: Postpartum depression.

T: Included studies that were published between Jan 1, 2000, and Sept 19, 2024.

### Search strategy

We performed a comprehensive literature search using PubMed, MEDLINE, Cochrane Library, EMBASE, and CINAHL databases to identify relevant peer-reviewed studies published in English between January 1, 2000, and September 19, 2024. Eligible studies had to examine PPD as an outcome and include prenatal exposure to nicotine (ATS, SHS, or ENP use) as an exposure or predictor.

We used the following search strategy using MeSH terms: ((((((((smoking[MeSH Terms]) OR (Nicotine[MeSH Terms])) OR (hookah[MeSH Terms])) OR (electronic cigarette[MeSH Terms])) OR (cigarette[MeSH Terms])) OR (tobacco[MeSH Terms])) OR (vape[MeSH Terms])) AND (pregnancy[MeSH Terms])) AND (postpartum depression[MeSH Terms]). We also used keywords with MeSH Terms Search (((((((((smoking[Title/Abstract]) OR (substance[Title/Abstract])) OR (nicotine[Title/Abstract])) OR (hookah[Title/Abstract])) OR (e-cigarette[Title/Abstract])) OR (cigarette[Title/Abstract])) OR (tobacco[Title/Abstract])) OR (vape[Title/Abstract])) AND (pregnancy[MeSH Terms])) AND (postpartum depression[MeSH Terms]). Search Terms were combined using the Boolean operator (AND, OR) with MeSH Term or searching Tile/Abstracts. Additionally, the references of the identified studies were manually searched to identify studies that might not have appeared in the original search.

### Inclusion and exclusion criteria

Studies were included if they met the following criteria: participants were pregnant or postpartum women (within 12 months); the study used an observational design (prospective or retrospective cohort, case-control, or cross-sectional); PPD was assessed as the primary outcome within the first year postpartum; and prenatal exposure to nicotine (ATS, SHS, or ENP) was assessed as an independent variable, covariate, or predictor. Eligible studies had to report an effect estimate (e.g., odds ratio, risk ratio, or prevalence ratio) or frequencies of exposure and outcome, include a comparison group (e.g., non-smokers), and be published in peer-reviewed indexed journals in English between (January 1, 2000, and September 19, 2024).

We excluded studies that examined smoking only before pregnancy or after childbirth; ecological studies, literature reviews, systematic reviews, or case series; studies without a comparison group; and those that reported perinatal depression without separating antenatal from postpartum outcomes. Full-text articles were reviewed to confirm eligibility and extract data.

### Study selection

All records identified through the database search were imported into EndNote and checked for duplication. After removing duplicates, titles and abstracts were screened for relevance. Full texts of potentially eligible studies were then reviewed to confirm inclusion. In cases where full texts or relevant data on PEN were missing, we contacted the corresponding authors. Two reviewers (R.Sh. and M.S.) independently performed the screening and selection process, and disagreements were resolved through discussion.

### Data extraction

Data were independently extracted by two researchers using a standardized form and cross-checked for consistency and accuracy. Extracted information included the study authors, year of publication, country, study population, study design, sample size, exposure definition and ascertainment method, comparison group, outcome definition ascertainment method, PPD prevalence, and crude and adjusted effect sizes (OR, RR, or PR) with 95% confidence intervals. For studies that presented data stratified by subgroups (e.g., age groups or exposure categories), each subgroup was included as a separate record in the meta-analysis. As a result, the number of data points in the meta-analysis may exceed the number of unique studies.

### Quality assessment

The quality of each included study was assessed using the Newcastle–Ottawa Scale (NOS), which is designed to evaluate the risk of bias in nonrandomized observational studies (GA Wells at al. 2021). The NOS has three versions tailored to different study designs: cohort studies, case-control studies, and an updated version for cross-sectional studies. In this systematic review, all three versions were utilized to match the design of the included studies. The scale evaluates study quality across three domains: selection of participants, comparability of groups, and exposure or outcome ascertainment. Then, scores were assigned for the items under each dimension. Each study was scored out of a maximum of nine points, with higher scores indicating higher quality. Based on total scores, studies were categorized as high quality (7–9 points), fair quality (2–6 points), or poor quality (< 2 points) (Mengist et al. 2021). Two reviewers (R.Sh. and M.S.) independently assessed study quality, and any disagreements were resolved by discussion.

### Statistical analysis

All statistical analyses were conducted using STATA version 17 (*StataCorp* LLC, College Station, TX) (StataCorp 2021). We calculated the pooled prevalence (PP) of PPD and the pooled odds ratio (pooled-OR) for the association between PEN and PPD. PP estimates were calculated using the *metaprop* command with Freeman-Tukey double arcsine transformation to stabilize variances. Pooled-OR and 95% confidence intervals were estimated using random-effects models (DerSimonian and Laird method) to account for between-studies heterogeneity. The significance of pooled estimates was tested using the Z test. Heterogeneity was assessed using Cochran’s Q test and the I² statistic, with thresholds of 0–25% for low, > 25–50% for moderate, and > 50% for high heterogeneity.

Subgroup analyses were conducted based on outcome ascertainment, study design, region, and study quality. Meta-regression explored the impact of study characteristics on the pooled OR. Publication bias was assessed using funnel plots and Egger’s and Begg’s tests. If bias was suspected, the trim-and-fill method estimated the potential influence of unpublished studies. Sensitivity analyses included leave-one-out analysis and restriction to only cohort studies, studies using EPDS or PRAMS, U.S.-based studies, and high-quality studies based on NOS scoring.

## Results

**Fig. 1:**
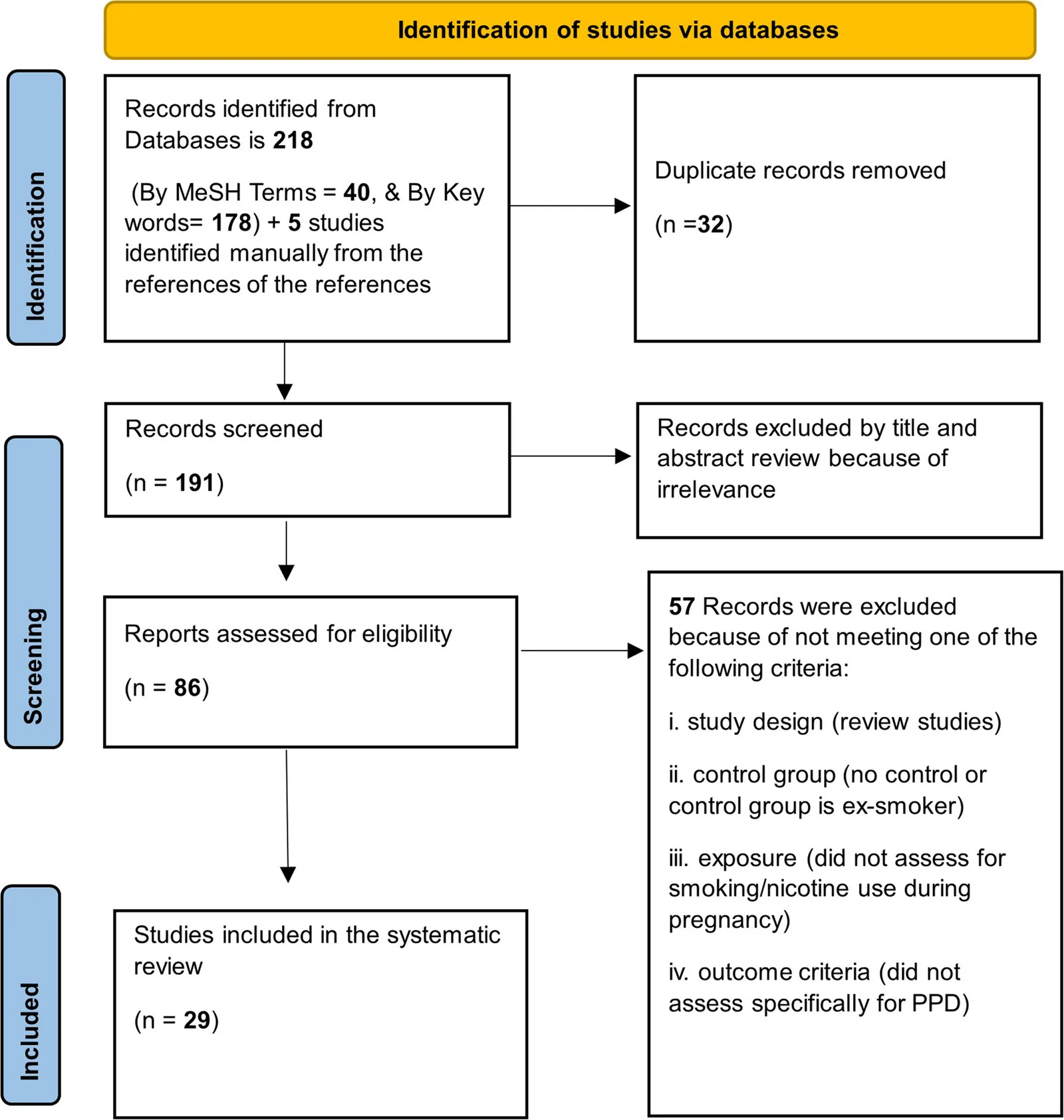
Flow chart showing the process of selection of the included studies in the systematic review

The initial search identified 218 studies (40 from MeSH terms and 178 from keyword searches), with five additional studies found through manual reference checks. After removing 32 duplicates, 191 studies were screened. Title and abstract screening excluded 105 studies. The remaining 86 underwent full-text assessment, excluding 57 for reasons such as ineligible exposure (e.g., smoking not during pregnancy), lack of a comparison group, outcome not specific to PPD, or ineligible study design. A total of 29 studies met the inclusion criteria for systematic review, and 26 were included in the meta-analysis (Fig. 1).

 Table 1 summarizes the characteristics of the included studies. Majority (19) were cross-sectional, (Alhammadi et al. 2023; Barber and Shenassa 2021; Choi et al. 2024; Cui et al. 2020; Farr et al. 2014; Haile et al. 2024; Harrison et al. 2023; Ho-Yen et al. 2007; Kang et al. 2022; Khan et al. 2015; Moore Simas et al. 2022; Nunes and Phipps 2013; Pooler et al. 2013; Shang et al. 2023; Silva et al. 2023; Sylvén et al. 2013; Tauqeer et al. 2023; Toru et al. 2018; Zejnullahu et al. 2021) followed by 7 cohort, (Dagher and Shenassa 2012; Jeon et al. 2024; Mora et al. 2009; Sidebottom et al. 2014; Song et al. 2019; van Lee et al. 2020; Wiener 2023) and 3 case-control designs. (Kondracki et al. 2024; Silva et al. 2021; Skalkidou et al. 2009) Nearly half (14) were conducted in the U.S. (with eight used PRAMS data, and the others used various data sources). (Barber and Shenassa 2021; Choi et al. 2024; Dagher and Shenassa 2012; Farr et al. 2014; Haile et al. 2024; Jeon et al. 2024; Khan et al. 2015; Kondracki et al. 2024; Moore Simas et al. 2022; Mora et al. 2009; Nunes and Phipps 2013; Pooler et al. 2013; Sidebottom et al. 2014; Wiener 2023) Seven studies were from Asia, (Cui et al. 2020; Kang et al. 2022; Shang et al. 2023; Song et al. 2019; van Lee et al. 2020); Alhammadi et al. 2023; Ho-Yen et al. 2007) five from Europe, (Harrison et al. 2023; Skalkidou et al. 2009; Sylvén et al. 2013; Tauqeer et al. 2023; Zejnullahu et al. 2021) two from South America, (Silva et al. 2021, 2023) and one from Africa. (Toru et al. 2018) PPD was assessed using a variety of tools, most commonly the EPDS (16 studies) with cutoffs ranging from (9–13); 8 used PRAMS questions, others include PHQ-9, CES-D, ICD-10, and diagnostic codes. The timing of PPD assessment ranged from 48 h to 12 months postpartum, most commonly between 2 and 6 months. 

**Table 1:** Summary of the 29 studies included in the systematic review

Author, year	Study Site	Aim of the study	Study population	Exposure definition	Exposure ascertainment Method	Study design	Comparison group
Gabriela A Barber et al., 2021	United States	To assess whether prenatal smoking predicts Postpartum Depression	Women who are 2–6 months postpartum during 2012–2015, the period of PRAMS data collection from 36 states in addition to New York City in the United States	ATS	Self-reported: Question in PRAMS data asking about smoking during the last 3 months of pregnancy	Cross-sectional	No smoking during pregnancy
Haile et al., 2024	United States	To assess the association between hookah smoking and postpartum depression	Women who are 2–6 months postpartum during 2016 - 2020, the period of PRAMS data collection in the United States	ATS (hookah)	Self-reported: Question in PRAMS (In the last two years before delivery: “Q: Have you used a hookah in the past 2 years?” This variable was coded as a dichotomous variable (yes/no).”	Cross-sectional	No Hookah use in the past two years
Nunes et al., 2013	United States	To assess the risk factors of PPD and assess whether these risk factors differ between adolescent and adults’ mothers	Women who are 2–6 months postpartum during 2004 - 2008, the period of PRAMS data collection in the in the Rhode Island in the United States	ATS	Self-reported: Question in PRAMS data asking about smoking during the last 3 months of pregnancy	Cross-sectional	No smoking during pregnancy
Harrison et al., 2023	United Kingdom	To assess and compare the association between different risk factors (including prenatal tobacco smoking) and postpartum depression before and during the pandemic	Women who give birth in England during 2014, 2018, & 2020	ATS	Self-reported	Cross-sectional	No smoking during pregnancy
Khan et al., 2015	United States	To assess the association between prenatal exposure to Second-hand smoking and PPD	Women who are 2–6 months postpartum during 2004 - 2008, the period of PRAMS data collection in the) in North Carolina in the United States	SHS	Self-reported: PRAMS question " How many cigarette smokers, not including yourself, lived in your home during your most recent pregnancy? answers " none, one, two or more smokers” (Second-hand smoking (Yes: ‘one’ or ‘two or more smokers’, No: none or zero smokers)	Cross-sectional	Not exposed to SHS
Choi et al., 2024	United States	To assess the association between e-cigarette use during pregnancy and PPD	Women who are 2–6 months postpartum during 2016 - 2019, the period of PRAMS data collection in the United States	ENP	Self-reported: PRAMS Question “During pregnancy asking about e-cigarette during the last 3 months of pregnancy"	Cross-sectional	No e-cigarette use during pregnancy
Cui et al. 2020	Japan	To assess the association between prenatal smoking and PPD among women in Japan	Women who were pregnant between January 2011 and March 2014 in Japan and eligible to participate in the Environment and Children’s Study (JECS),	ATS	Self-reported: assessed by self-administered questionnaires at second/third trimester	Cross-sectional	No smoking during pregnancy
Dagher et al., 2012	United States	To investigate the association between cigarette smoking, caffeine intake, and vitamin intake during pregnancy and PPD at 8 weeks after childbirth	All women delivering at birthing hospital at northeastern city between 2005 to 2008	ATS & SHS	Self-reported: data collected by interview, participants were asked about smoking cigarettes or exposure to second hand smoking during pregnancy	Cohort	No cigarette smoking/no exposure to SHS
Ho-Yen et al., 2007	Nepal	To explore risk factors for postnatal depression in different settings in Nepal (clinic, urban, rural areas)	Women who give birth in Patan City, Nepal	ATS	Self-reported	Cross-sectional	No smoking during pregnancy
Tauqeer et al., 2023	Belgium, Norway, Switzerland, the Netherlands and the UK	To identify risk factors related to depressive and anxiety symptoms among pregnant and postpartum women.	Pregnant women and women up to 3 months postpartum in Belgium, Norway, Switzerland, the Netherlands, and the UK between 10 June 2021 and 22 August 2021	ATS	Self-reported: assessed by online survey	Cross-sectional	No smoking during pregnancy
Simas et al., 2022	United States	to identify sociodemographic, behavioral, psychosocial, & medical factors associated with PPD among Puerto Rican women with pregnancy hyperglycemia	Puerto Rican Hispanic women who have pregnancy hyperglycemia and participated in the Estudio Parto randomized controlled trial conducted Jan 2013 - Dec. 2017	ATS	Self-reported	Cross-sectional	No smoking during pregnancy
Kang et al., 2022	South Korea	To investigate the prevalence of PPD and its prenatal risk factors	All pregnant women in Seoul who were a sample of women participated in the Healthy First Step Project in 2013, South Korea	ATS	Self-reported: data collected during pregnancy: do you smoke currently?” (Yes/No)	Cross-sectional	No smoking during pregnancy
Zejnullahu et al., 2021	Kosovo	To examine the prevalence and risk factors of PPD in Kosovo	Women attended clinics of Obstetrics & Gynecology in Pristina, Kosovo between June to Oct. 2019	ATS	Self-reported in face-to-face interview	Cross-sectional	No smoking during pregnancy
Lee et al., 2020	Singapore	To investigate the cumulative risk of lifestyle behaviors on depressive symptoms during pregnancy and after delivery	Pregnant women in Singapore during the period from June 2009 to September 2010 who participated in the Growing Up in Singapore Towards Healthy Outcomes cohort study	ATS	Self-reported: assessed during the antenatal clinic visits.	Cohort	No smoking during pregnancy
Toru et al., 2018	Ethiopia	To assess the magnitude and risk factors of PPD among postpartum women in Ethiopia	Populations for the study were all postpartum women who gave birth within 12 months before data collection (15 March- 15 April 2017) in Ethiopia	ATS	Self-reported: assessed as part of substance use during pregnancy by face to face interview using structured questionnaires.	Cross-sectional	No substance use during pregnancy
Farr et al., 2014	United States	To estimate the prevalence and risk factors of anxiety and comorbid depressive symptoms of postpartum women	Women who are 2–6 months postpartum during 2009 - 2010, the period of PRAMS data collection in the in Illinois and Maryland, United States	ATS	Self-reported	Cross-sectional	No smoking during pregnancy
Sidebottom et al., 2014	United States	To assess and examine the risk factors of depressive symptoms at prenatal, postpartum, or at both periods	Women of reproductive age who are enrolled in Healthy Start Initiative targets funding to communities with high rates of infant mortality at Minneapolis in Minnesota between November 2005 and May 2009.	ATS	Self-reported: assessed with two questions: frequency and average number of cigarettes smoked per day. If smoking during pregnancy (moderate/high risk), no smoking during pregnancy (No/low risk)	Cohort	No smoking during pregnancy
Pooler et al., 2013	United States	To determine the prevalence and risk factors of PPD among women enrolled or eligible to WIC program	Women who are 2–6 months postpartum during 2006 - 2008, the period of PRAMS data collection in the United States	ATS	Self-reported: data collected after pregnancy, structured questionnaire	Cross-sectional	No smoking during pregnancy
Sylvén et al., 2013	Sweden	To assess the association between thyroid hormone level at the time of delivery and PPD.	Women who gave birth in the county of Uppsala, Sweden, between May 21, 2006, to June 26, 2007	ATS	Self-reported by structured questionnaire	Cross-sectional	No smoking during pregnancy
Skalkidou et al., 2009	Sweden	To investigate the association between leptin and IL-6 levels around the time of delivery and postpartum depression	This is a case-control study nested in a cohort study. Therefore, the study population is the participants of the cohort study (women participated in UPPSAT project between November 2006 to May 2007, a population-based cohort study in the county of Uppsala, Sweden, investigating correlates of postpartum depression)	ATS	Self-reported by structured questionnaire	Case-control	No smoking during pregnancy
Kondracki et al., 2024	United States	To investigate the association between maternal smoking pattern and PPD and whether antenatal depression modify this association	Women who gave birth in the US during 2017 - 2018 the period of data collection for PRAMS	ATS	Self-reported by structured questionnaire	Case-control	No smoking during pregnancy
Alhammadi et al., 2023	Saudia Arabia	to assess the association between PPD and risk factors, such as delivery mode, ABO blood group, and passive smoking in Saudi Arabia.	Women who had been pregnant and given birth within between Jan - March 2022 in Saudi Arabia	ATS & SHS	Self-reported by structured questionnaire	Cross-sectional	No Secondhand smoking during pregnancy, No smoking during pregnancy
Jeon et al., 2024	United States	to estimate the prevalence of diagnosed PPD and investigate the maternal risk factors of PPD and whether these risk factors varies by maternal age (adolescents’ mothers vs. adults)	Women who had their first live birth between 2008 and 2015 women in Utah, United States	ATS	Self-reported by structured questionnaire	Cohort	No tobacco smoking during pregnancy
Wiener et al., 2023	United States	To examine the association of nicotine dependence any time prior to delivery and PPD	Data from TriNetX Research Network platform of pregnant women during postpartum period	Nicotine Dependence	Self-reported by structured questionnaire	Cohort	No Nicotine dependence
Oliveira et al., 2023	Brazil	to investigate the association between postpartum depression symptoms and anxiety and stress symptoms in the postpartum period.	Women aged 18 years or older receiving care at a public maternity hospital in a metropolitan city in Brazil between June 2021 and February 2022.	ATS	Self-reported by structured questionnaire	Cross-sectional	No tobacco use during pregnancy
Song et al. 2019	China	To assess whether active or Secondhand smoking is associated with PPD	Women who gave birth between 2010 - 2012 in Tianjin, China " Women who were registered in Maternal and Child Health Information System”.	ATS & SHS	Self-reported	Cohort	No active smoking, no Secondhand smoking during pregnancy
Silva et al., 2021	Brazile	To investigate and identify risk factors of postpartum depression and correlate postpartum depression with maternal anxiety among women in Brazil.	Women who gave birth during 2017 and 2018 in the city of Vila Velha, Espirito Santo, Brazil	ATS	Self-reported using structured questionnaire	Case-control	No smoking during pregnancy
Shang et al., 2023	China	To explore the independent and combined effects of smoking and passive smoking during pregnancy on maternal depression, anxiety and depressive anxiety comorbidities.	Women who underwent 42-day postpartum examination in Hospital of Hefei	ATS and SHS	Self-reported using structured questionnaire	Cross-sectional	No active or secondhand smoking during pregnancy
Mora et al., 2009	United States	To identify trajectory classes of depressive symptoms from prenatal to postpartum periods and investigate the demographic, socioeconomic, health, health behavior, and psychosocial characteristics of antenatal and postpartum depression	Women of childbearing age in low-income, inner-city women in Philadelphia, Pennsylvania.	ATS	Self-reported	Cohort	No active tobacco smoking during pregnancy
Author, year	Outcome definition	Outcome ascertainment method	Sample size	PPD prevalence	RR Crude	RR Adjusted	Findings of the study
Gabriela A Barber et al., 2021	PPD (Yes/No)	Self-reported: PRAMS questions (Q1: How often since your baby was born do you feel down, depressed or hopeless & Q2: How often since your baby was born do you had little interest or pleasure in doing things). Answers a 5-point Likert scale (“Always”, “Often”, “Sometimes”, “Rarely”, or “Never”). PPD (Yes: “Always”, “Often”/No: “Sometimes”, “Rarely”, or “Never”)	134435	11.50%	OR: 1.95 95%CI: 1.49 - 2.56	OR: 1.41; 95% CI: 1.06 - 1.86	Significant association between prenatal ATS and PPD
Haile et al., 2024	PPD (Yes/No)	Self-reported: PRAMS questions (Q1: How often since your baby was born do you feel down, depressed or hopeless & Q2: How often since your baby was born do you had little interest or pleasure in doing things). Answers a 5-point Likert scale (“Always”, “Often”, “Sometimes”, “Rarely”, or “Never”). PPD (Yes: “Always”, “Often”/No: “Sometimes”, “Rarely”, or “Never”)	106894	8.20%		OR: 1.20; 95%CI: 1.03 - 1.40	Significant association between hookah use and PPD
Nunes et al., 2013	PPD (Sever - Moderate/Mild/No).	Self-reported: PRAMS questions (Q1: How often since your baby was born do you feel down, depressed or hopeless & Q2: How often since your baby was born do you had little interest or pleasure in doing things). Answers a 5-point Likert scale (“Always”, “Often”, “Sometimes”, “Rarely”, or “Never”). PPD (Yes: “Always”, “Often”/No: “Sometimes”, “Rarely”, or “Never”). Sum score from both questions (PPD: moderate - sever > 5 sum score, mild 3 - 5, No < 3)	6,959	(age group 15 - 19): 12.11%	OR: 2.06; 95%CI: 0.97 - 4.38		Significant association between prenatal ATS and PPD in age group (20 - 24), but not among adolescents and females older than 30
				(age group 20 - 24) 9.77%	OR: 1.98; 95%CI: 1.16 - 3.39		
				(age group 25 - 29):7.32%	OR: 1.20; 95%CI: 0.59 - 2.44		
				(age group 30+): 4.78%	OR: 1.39; 95%CI: 0.64 - 3.01		
Harrison et al., 2023	PPD (Yes/No)	Self-reported: assessed Edinburgh Postnatal Depression Scale (EPDS)≥ 13	32,050	2014: 10.3%	Prenatal tobacco smoking was not assessed in the survey of 2014	OR: 1.43; 95%: 1.13 - 1.80	Significant association between prenatal ATS and PPD among participants during 2018, but not during the year 2020
				2018: 16%	2018: OR 2.03; 95%CI: 1.63 - 2.54		
				2020: 23.9%	2020: OR: 1·67; 95%CI:1·38 - 2·02		
Khan et al., 2015	PPD (Yes/No)	Self-reported: PRAMS questions (Q1: How often since your baby was born do you feel down, depressed or hopeless & Q2: How often since your baby was born do you had little interest or pleasure in doing things). Answers a 5-point Likert scale (“Always”, “Often”, “Sometimes”, “Rarely”, or “Never”). PPD (Yes: “Always”, “Often”/No: “Sometimes”, “Rarely”, or “Never”)	6884	16.52%	OR: 1.90; 95% CI: 1.61–2.26	OR: 1.49; 95% CI: 1.23–1.80	Significant association between prenatal SHS and PPD
Choi et al., 2024	PPD (Yes/No).	Self-reported: PRAMS questions (Q1: How often since your baby was born do you feel down, depressed or hopeless & Q2: How often since your baby was born do you had little interest or pleasure in doing things). Answers a 5-point Likert scale (“Always”, “Often”, “Sometimes”, “Rarely”, or “Never”). PPD (Yes: “Always”, “Often”/No: “Sometimes”, “Rarely”, or “Never”)	58950	13.34%	1.14 (0.82–1.58)	OR: 1.03; 95%CI: 0.73–1.46	No significant association between prenatal Electronic Nicotine Products use (e-cig) and PPD
Cui et al. 2020	PPD (Yes/No)	Self-reported: measured by EPDS (PPD ≥9 score in EPDS)	80,872	9%	OR=1.64 (95%CI:1.47–1.83)	OR:1.38; 95%CI:1.21–1.56	Significant association between prenatal ATS and PPD
Dagher et al., 2012	PPD (Yes/No)	Self-reported: measured by Edinburgh Postnatal Depression Scale through interview at 8 weeks after child birth using structured questionnaire	662	6.50%	(Active smoking Regression Coefficient: 2.34), (SHS Regression Coefficient: 0.02)	(Active smoking Regression Coefficient: 1.74), (SHS Regression Coefficient: 0.02)	Significant association between prenatal ATS and PPD, but No significant association between prenatal SHS and PPD
Ho-Yen et al., 2007	PPD (Yes/No)	Self-reported: assessed by EPDS (PPD >12score EPDS) through structured interview	426	4.90%	6.6 [1.9 - 22]	5.6 (1.2 - 25.6)	Significant association between prenatal ATS and PPD
Tauqeer et al., 2023	PPD (Yes/No)	Self-reported: assessed by Edinburgh Postanal Depression Scale (EPDS) PPD is ≥13 score	1799	17%	1.47 (0.62 to 3.44)		No significant association between prenatal ATS and PPD
Simas et al., 2022	PPD (Yes/No)	Self-reported: assessed by EPDS (PPD≥ 10 score of EPDS)	203	27%		OR=2.96 (95%CI: 1.41 - 6.18)	Significant association between prenatal ATS and PPD
Kang et al., 2022	PPD (Yes/No)	Self-reported: : assessed by Korean version of EPDS (PPD ≥ 10 score of EPDS)	80116	24.30%	7.34 (5.64–9.56)	2.02 (1.44–2.83)	Significant association between prenatal ATS and PPD
Zejnullahu et al., 2021	PPD (Yes/No)	Self-reported: assessed by face to face interview using Albanian translated EPDS. (PPD ≥12 EPDS score)	247	21%	1.088 (0.997–1.187)		No significant association between prenatal ATS and PPD
Lee et al., 2020	PPD (Yes/No)	Self-reported: assessed by EPDS at 3 months after delivery (PPD ≥13 EPDS score).	356 (the original sample size was 1247, after excluding those missing data about either EPDS or life style risk factors, only 356 were included in this analysis)				No significant association between ATS and PPD
Toru et al., 2018	PPD (Yes/No)	Self-reported: assessed by interview using 9-items PHQ9	456	22.40%		5.17, (95%CI: 2.52–10.60)	Significant association between prenatal ATS and PPD
Farr et al., 2014	PPD (Yes/No)	Self-reported: PRAMS questions (Q1: How often since your baby was born do you feel down, depressed or hopeless & Q2: How often since your baby was born do you had little interest or pleasure in doing things). Answers a 5-point Likert scale (“Always”, “Often”, “Sometimes”, “Rarely”, or “Never”). PPD (Yes: “Always”, “Often”/No: “Sometimes”, “Rarely”, or “Never”)	4451	9%		OR= 1.4; 95%CI: 0.7 - 2.7	No significant association between ATS and PPD
Sidebottom et al., 2014	PPD (Yes/No)	Self-reported: assessed by PHQ-9 questions (each question has answers scored 0 - 3) the scores of all questions were then summed (PPD≥ 10)	594	6%		OR: 1.36 (95%CI:0.56 - 3.31)	Significant association between prenatal ATS and PPD
Pooler et al., 2013	PPD (Yes/No)	Self-reported: PRAMS questions (Q1: How often since your baby was born do you feel down, depressed or hopeless & Q2: How often since your baby was born do you had little interest or pleasure in doing things). Answers a 5-point Likert scale (“Always”, “Often”, “Sometimes”, “Rarely”, or “Never”). PPD (Yes: “Always”, “Often”/No: “Sometimes”, “Rarely”, or “Never”)	75,234	Overall prevalence (WIC and non-WIC: 13.8%), prevalence among WIC participants: 19.8%, prevalence among WIC eligible but not participants: 16.3%, prevalence among women not eligible to WIC: 6.8%	Overall OR: 1.65 (95%CI: 1.46 - 1.85)	OR: 1.59 (95%CI: 1.42 - 1.79)	Significant association between prenatal ATS and PPD
Sylvén et al., 2013	PPD (Yes/No)	Self-reported: assessed by structured questionnaire and measured by EPDS. (PPD≥12 EPDS score)	329	11.70%	OR: 1.73 (95%CI: 0.13 - 22.53)	OR: 1.57 (0.08 - 29.12)	No significant association between prenatal ATS and PPD
Skalkidou et al., 2009	PPD (Yes/No)	Self-reported: Assessed by EPDS (the Swedish version) the cutoff point is ≥ 12 EPDS score.	347	This is a case control study		linear regression coefficient: 0.87	No significant association between prenatal ATS and PPD
Kondracki et al., 2024	PPD (Yes/No)	Self-reported: PRAMS questions (Q1: How often since your baby was born do you feel down, depressed or hopeless & Q2: How often since your baby was born do you had little interest or pleasure in doing things). Answers a 5-point Likert scale (“Always”, “Often”, “Sometimes”, “Rarely”, or “Never”). PPD (Yes: “Always”, “Often”/No: “Sometimes”, “Rarely”, or “Never”)	51220	This is a case control study		Low-intensity smokers (1st &2nd Trimester): OR 1.22(95CI%: 1.18 - 1.25)	Significant association between prenatal ATS and PPD
						High-intensity smoker (1st&2nd Trimester): OR=1.21 (95%CI: 1.17 - 1.26)	
						All trimesters (low-intensity): OR=1.17 (95%CI: 1.15 - 1.19)	
						All trimesters (high-intensity): OR=1.165 (95%CI: 1.63 - 1.68)	
						Reduced smoking towards the end of pregnancy: OR=1.13 (95%CI: 1.10 - 1.17)	
						Increased towards the end of pregnancy: OR=0.58 (95%CI: 0.45 - 0.74)	
Alhammadi et al., 2023	PPD (Yes/No)	Assessed by EPDS this scale questions were translated into Arabic and bac translated into English by two independent authors	354	56.20%	0.78		No significant association between prenatal ATS and PPD, and No significant association between prenatal SHS and PPD
Jeon et al., 2024	PPD (Yes/No)	Defined as depression-related diagnostic code “depression-related diagnosis codes was based on Clinical Classification Software (CCS) provided by the Agency for Healthcare Research and Quality (AHRQ).”	61,226	Overall prevalence: 4.04%, (PPD in adults: 3.8%), (PPD in adolescents: 6.08%)	(OR: 1.28 [95% CI: 1.12–1.41] in adults; OR: 1.86 [95% CI: 1.40–2.47] in adolescents)		Significant association between prenatal ATS and PPD
Wiener et al., 2023	PPD (Yes/No)	ICD 10 code	10598		(RR: 1.75 [95% CI: 1.55, 1.9] in unmatched data; RR: 0.68 [95% CI: 0.59, 0.79] matching upon age, race, supervision of high-risk pregnancy, gestational diabetes mellitus, diabetes mellitus, mental/behavioral/neurodevelopmental disorder, person with potential health hazards related to socioeconomic status and psychosocial concerns, preterm (premature) newborn, extreme immaturity of newborn and another low birthweight newborn)	1.75	Significant association between nicotine dependence and PPD
Oliveira et al., 2023	PPD (Yes/No)	Assessed using the Edinburgh Postpartum Depression Scale (EPDS). PPD ≥ 13 EPDS	101	24.80%	3.02 (1.35–8.79)	2.15 (1.51–4.66)	Significant association between prenatal ATS and PPD
Song et al. 2019	PPD (Yes/No)	Assessed by Chines version of EPDS. PPD ≥ 10 EPDS	8842	8.50%	Active smoking 1.00 (95%CI: 0.83–1.18), passive smoking 1.13 (95% CI: 1.02–1.26)	1.43, (95 CI: 1.16–1.77)	Significant association between prenatal SHS and PPD, No significant association between prenatal ATS and PPD
Silva et al., 2021	PPD (Yes/No)	Assessed by EPDS > 10 score	227		1.08 (0.57 - 2.03)		No significant association between prenatal ATS and PPD
Shang et al., 2023	PPD(Yes/No)	Assessed by EPDS	2447	14.80%		(Active smoking: OR=3.86, 95%CI 2.37–6.28) & (Passive smoking: OR = 1.56, 95%CI 2.00–5.71)	Significant association between prenatal SHS and PPD, and significant association between prenatal ATS and PPD
Mora et al., 2009	PPD (Yes/No)	Measured utilizing the Center for Epidemiologic Studies Depression (CES-D) Scale. Cutoff points of ≥16 and ≥23 score indicating significant levels of depressive symptomatology	1735	9%	OR =1.09	OR = 0.92 (0.85 - 1.45)	No significant association between prenatal ATS and PPD
Author, year	Study Site	Aim of the study	Study population	Exposure definition	Exposure ascertainment Method	Study design	Comparison group
Gabriela A Barber et al., 2021	United States	To assess whether prenatal smoking predicts Postpartum Depression	Women who are 2–6 months postpartum during 2012–2015, the period of PRAMS data collection from 36 states in addition to New York City in the United States	ATS	Self-reported: Question in PRAMS data asking about smoking during the last 3 months of pregnancy	Cross-sectional	No smoking during pregnancy
Haile et al., 2024	United States	To assess the association between hookah smoking and postpartum depression	Women who are 2–6 months postpartum during 2016 - 2020, the period of PRAMS data collection in the United States	ATS (hookah)	Self-reported: Question in PRAMS (In the last two years before delivery: “Q: Have you used a hookah in the past 2 years?” This variable was coded as a dichotomous variable (yes/no).”	Cross-sectional	No Hookah use in the past two years
Nunes et al., 2013	United States	To assess the risk factors of PPD and assess whether these risk factors differ between adolescent and adults’ mothers	Women who are 2–6 months postpartum during 2004 - 2008, the period of PRAMS data collection in the in the Rhode Island in the United States	ATS	Self-reported: Question in PRAMS data asking about smoking during the last 3 months of pregnancy	Cross-sectional	No smoking during pregnancy
Harrison et al., 2023	United Kingdom	To assess and compare the association between different risk factors (including prenatal tobacco smoking) and postpartum depression before and during the pandemic	Women who give birth in England during 2014, 2018, & 2020	ATS	Self-reported	Cross-sectional	No smoking during pregnancy
Khan et al., 2015	United States	To assess the association between prenatal exposure to Second-hand smoking and PPD	Women who are 2–6 months postpartum during 2004 - 2008, the period of PRAMS data collection in the) in North Carolina in the United States	SHS	Self-reported: PRAMS question " How many cigarette smokers, not including yourself, lived in your home during your most recent pregnancy? answers " none, one, two or more smokers” (Second-hand smoking (Yes: ‘one’ or ‘two or more smokers’, No: none or zero smokers)	Cross-sectional	Not exposed to SHS
Choi et al., 2024	United States	To assess the association between e-cigarette use during pregnancy and PPD	Women who are 2–6 months postpartum during 2016 - 2019, the period of PRAMS data collection in the United States	ENP	Self-reported: PRAMS Question “During pregnancy asking about e-cigarette during the last 3 months of pregnancy"	Cross-sectional	No e-cigarette use during pregnancy
Cui et al. 2020	Japan	To assess the association between prenatal smoking and PPD among women in Japan	Women who were pregnant between January 2011 and March 2014 in Japan and eligible to participate in the Environment and Children’s Study (JECS),	ATS	Self-reported: assessed by self-administered questionnaires at second/third trimester	Cross-sectional	No smoking during pregnancy
Dagher et al., 2012	United States	To investigate the association between cigarette smoking, caffeine intake, and vitamin intake during pregnancy and PPD at 8 weeks after childbirth	All women delivering at birthing hospital at northeastern city between 2005 to 2008	ATS & SHS	Self-reported: data collected by interview, participants were asked about smoking cigarettes or exposure to second hand smoking during pregnancy	Cohort	No cigarette smoking/no exposure to SHS
Ho-Yen et al., 2007	Nepal	To explore risk factors for postnatal depression in different settings in Nepal (clinic, urban, rural areas)	Women who give birth in Patan City, Nepal	ATS	Self-reported	Cross-sectional	No smoking during pregnancy
Tauqeer et al., 2023	Belgium, Norway, Switzerland, the Netherlands and the UK	To identify risk factors related to depressive and anxiety symptoms among pregnant and postpartum women.	Pregnant women and women up to 3 months postpartum in Belgium, Norway, Switzerland, the Netherlands, and the UK between 10 June 2021 and 22 August 2021	ATS	Self-reported: assessed by online survey	Cross-sectional	No smoking during pregnancy
Simas et al., 2022	United States	to identify sociodemographic, behavioral, psychosocial, & medical factors associated with PPD among Puerto Rican women with pregnancy hyperglycemia	Puerto Rican Hispanic women who have pregnancy hyperglycemia and participated in the Estudio Parto randomized controlled trial conducted Jan 2013 - Dec. 2017	ATS	Self-reported	Cross-sectional	No smoking during pregnancy
Kang et al., 2022	South Korea	To investigate the prevalence of PPD and its prenatal risk factors	All pregnant women in Seoul who were a sample of women participated in the Healthy First Step Project in 2013, South Korea	ATS	Self-reported: data collected during pregnancy: do you smoke currently?” (Yes/No)	Cross-sectional	No smoking during pregnancy
Zejnullahu et al., 2021	Kosovo	To examine the prevalence and risk factors of PPD in Kosovo	Women attended clinics of Obstetrics & Gynecology in Pristina, Kosovo between June to Oct. 2019	ATS	Self-reported in face-to-face interview	Cross-sectional	No smoking during pregnancy
Lee et al., 2020	Singapore	To investigate the cumulative risk of lifestyle behaviors on depressive symptoms during pregnancy and after delivery	Pregnant women in Singapore during the period from June 2009 to September 2010 who participated in the Growing Up in Singapore Towards Healthy Outcomes cohort study	ATS	Self-reported: assessed during the antenatal clinic visits.	Cohort	No smoking during pregnancy
Toru et al., 2018	Ethiopia	To assess the magnitude and risk factors of PPD among postpartum women in Ethiopia	Populations for the study were all postpartum women who gave birth within 12 months before data collection (15 March- 15 April 2017) in Ethiopia	ATS	Self-reported: assessed as part of substance use during pregnancy by face to face interview using structured questionnaires.	Cross-sectional	No substance use during pregnancy
Farr et al., 2014	United States	To estimate the prevalence and risk factors of anxiety and comorbid depressive symptoms of postpartum women	Women who are 2–6 months postpartum during 2009 - 2010, the period of PRAMS data collection in the in Illinois and Maryland, United States	ATS	Self-reported	Cross-sectional	No smoking during pregnancy
Sidebottom et al., 2014	United States	To assess and examine the risk factors of depressive symptoms at prenatal, postpartum, or at both periods	Women of reproductive age who are enrolled in Healthy Start Initiative targets funding to communities with high rates of infant mortality at Minneapolis in Minnesota between November 2005 and May 2009.	ATS	Self-reported: assessed with two questions: frequency and average number of cigarettes smoked per day. If smoking during pregnancy (moderate/high risk), no smoking during pregnancy (No/low risk)	Cohort	No smoking during pregnancy
Pooler et al., 2013	United States	To determine the prevalence and risk factors of PPD among women enrolled or eligible to WIC program	Women who are 2–6 months postpartum during 2006 - 2008, the period of PRAMS data collection in the United States	ATS	Self-reported: data collected after pregnancy, structured questionnaire	Cross-sectional	No smoking during pregnancy
Sylvén et al., 2013	Sweden	To assess the association between thyroid hormone level at the time of delivery and PPD.	Women who gave birth in the county of Uppsala, Sweden, between May 21, 2006, to June 26, 2007	ATS	Self-reported by structured questionnaire	Cross-sectional	No smoking during pregnancy
Skalkidou et al., 2009	Sweden	To investigate the association between leptin and IL-6 levels around the time of delivery and postpartum depression	This is a case-control study nested in a cohort study. Therefore, the study population is the participants of the cohort study (women participated in UPPSAT project between November 2006 to May 2007, a population-based cohort study in the county of Uppsala, Sweden, investigating correlates of postpartum depression)	ATS	Self-reported by structured questionnaire	Case-control	No smoking during pregnancy
Kondracki et al., 2024	United States	To investigate the association between maternal smoking pattern and PPD and whether antenatal depression modify this association	Women who gave birth in the US during 2017 - 2018 the period of data collection for PRAMS	ATS	Self-reported by structured questionnaire	Case-control	No smoking during pregnancy
Alhammadi et al., 2023	Saudia Arabia	to assess the association between PPD and risk factors, such as delivery mode, ABO blood group, and passive smoking in Saudi Arabia.	Women who had been pregnant and given birth within between Jan - March 2022 in Saudi Arabia	ATS & SHS	Self-reported by structured questionnaire	Cross-sectional	No Secondhand smoking during pregnancy, No smoking during pregnancy
Jeon et al., 2024	United States	to estimate the prevalence of diagnosed PPD and investigate the maternal risk factors of PPD and whether these risk factors varies by maternal age (adolescents’ mothers vs. adults)	Women who had their first live birth between 2008 and 2015 women in Utah, United States	ATS	Self-reported by structured questionnaire	Cohort	No tobacco smoking during pregnancy
Wiener et al., 2023	United States	To examine the association of nicotine dependence any time prior to delivery and PPD	Data from TriNetX Research Network platform of pregnant women during postpartum period	Nicotine Dependence	Self-reported by structured questionnaire	Cohort	No Nicotine dependence
Oliveira et al., 2023	Brazil	to investigate the association between postpartum depression symptoms and anxiety and stress symptoms in the postpartum period.	Women aged 18 years or older receiving care at a public maternity hospital in a metropolitan city in Brazil between June 2021 and February 2022.	ATS	Self-reported by structured questionnaire	Cross-sectional	No tobacco use during pregnancy
Song et al. 2019	China	To assess whether active or Secondhand smoking is associated with PPD	Women who gave birth between 2010 - 2012 in Tianjin, China " Women who were registered in Maternal and Child Health Information System”.	ATS & SHS	Self-reported	Cohort	No active smoking, no Secondhand smoking during pregnancy
Silva et al., 2021	Brazile	To investigate and identify risk factors of postpartum depression and correlate postpartum depression with maternal anxiety among women in Brazil.	Women who gave birth during 2017 and 2018 in the city of Vila Velha, Espirito Santo, Brazil	ATS	Self-reported using structured questionnaire	Case-control	No smoking during pregnancy
Shang et al., 2023	China	To explore the independent and combined effects of smoking and passive smoking during pregnancy on maternal depression, anxiety and depressive anxiety comorbidities.	Women who underwent 42-day postpartum examination in Hospital of Hefei	ATS and SHS	Self-reported using structured questionnaire	Cross-sectional	No active or secondhand smoking during pregnancy
Mora et al., 2009	United States	To identify trajectory classes of depressive symptoms from prenatal to postpartum periods and investigate the demographic, socioeconomic, health, health behavior, and psychosocial characteristics of antenatal and postpartum depression	Women of childbearing age in low-income, inner-city women in Philadelphia, Pennsylvania.	ATS	Self-reported	Cohort	No active tobacco smoking during pregnancy
Author, year	Outcome definition	Outcome ascertainment method	Sample size	PPD prevalence	RR Crude	RR Adjusted	Findings of the study
Gabriela A Barber et al., 2021	PPD (Yes/No)	Self-reported: PRAMS questions (Q1: How often since your baby was born do you feel down, depressed or hopeless & Q2: How often since your baby was born do you had little interest or pleasure in doing things). Answers a 5-point Likert scale (“Always”, “Often”, “Sometimes”, “Rarely”, or “Never”). PPD (Yes: “Always”, “Often”/No: “Sometimes”, “Rarely”, or “Never”)	134435	11.50%	OR: 1.95 95%CI: 1.49 - 2.56	OR: 1.41; 95% CI: 1.06 - 1.86	Significant association between prenatal ATS and PPD
Haile et al., 2024	PPD (Yes/No)	Self-reported: PRAMS questions (Q1: How often since your baby was born do you feel down, depressed or hopeless & Q2: How often since your baby was born do you had little interest or pleasure in doing things). Answers a 5-point Likert scale (“Always”, “Often”, “Sometimes”, “Rarely”, or “Never”). PPD (Yes: “Always”, “Often”/No: “Sometimes”, “Rarely”, or “Never”)	106894	8.20%		OR: 1.20; 95%CI: 1.03 - 1.40	Significant association between hookah use and PPD
Nunes et al., 2013	PPD (Sever - Moderate/Mild/No).	Self-reported: PRAMS questions (Q1: How often since your baby was born do you feel down, depressed or hopeless & Q2: How often since your baby was born do you had little interest or pleasure in doing things). Answers a 5-point Likert scale (“Always”, “Often”, “Sometimes”, “Rarely”, or “Never”). PPD (Yes: “Always”, “Often”/No: “Sometimes”, “Rarely”, or “Never”). Sum score from both questions (PPD: moderate - sever > 5 sum score, mild 3 - 5, No < 3)	6,959	(age group 15 - 19): 12.11%	OR: 2.06; 95%CI: 0.97 - 4.38		Significant association between prenatal ATS and PPD in age group (20 - 24), but not among adolescents and females older than 30
				(age group 20 - 24) 9.77%	OR: 1.98; 95%CI: 1.16 - 3.39		
				(age group 25 - 29):7.32%	OR: 1.20; 95%CI: 0.59 - 2.44		
				(age group 30+): 4.78%	OR: 1.39; 95%CI: 0.64 - 3.01		
Harrison et al., 2023	PPD (Yes/No)	Self-reported: assessed Edinburgh Postnatal Depression Scale (EPDS)≥ 13	32,050	2014: 10.3%	Prenatal tobacco smoking was not assessed in the survey of 2014	OR: 1.43; 95%: 1.13 - 1.80	Significant association between prenatal ATS and PPD among participants during 2018, but not during the year 2020
				2018: 16%	2018: OR 2.03; 95%CI: 1.63 - 2.54		
				2020: 23.9%	2020: OR: 1·67; 95%CI:1·38 - 2·02		
Khan et al., 2015	PPD (Yes/No)	Self-reported: PRAMS questions (Q1: How often since your baby was born do you feel down, depressed or hopeless & Q2: How often since your baby was born do you had little interest or pleasure in doing things). Answers a 5-point Likert scale (“Always”, “Often”, “Sometimes”, “Rarely”, or “Never”). PPD (Yes: “Always”, “Often”/No: “Sometimes”, “Rarely”, or “Never”)	6884	16.52%	OR: 1.90; 95% CI: 1.61–2.26	OR: 1.49; 95% CI: 1.23–1.80	Significant association between prenatal SHS and PPD
Choi et al., 2024	PPD (Yes/No).	Self-reported: PRAMS questions (Q1: How often since your baby was born do you feel down, depressed or hopeless & Q2: How often since your baby was born do you had little interest or pleasure in doing things). Answers a 5-point Likert scale (“Always”, “Often”, “Sometimes”, “Rarely”, or “Never”). PPD (Yes: “Always”, “Often”/No: “Sometimes”, “Rarely”, or “Never”)	58950	13.34%	1.14 (0.82–1.58)	OR: 1.03; 95%CI: 0.73–1.46	No significant association between prenatal Electronic Nicotine Products use (e-cig) and PPD
Cui et al. 2020	PPD (Yes/No)	Self-reported: measured by EPDS (PPD ≥9 score in EPDS)	80,872	9%	OR=1.64 (95%CI:1.47–1.83)	OR:1.38; 95%CI:1.21–1.56	Significant association between prenatal ATS and PPD
Dagher et al., 2012	PPD (Yes/No)	Self-reported: measured by Edinburgh Postnatal Depression Scale through interview at 8 weeks after child birth using structured questionnaire	662	6.50%	(Active smoking Regression Coefficient: 2.34), (SHS Regression Coefficient: 0.02)	(Active smoking Regression Coefficient: 1.74), (SHS Regression Coefficient: 0.02)	Significant association between prenatal ATS and PPD, but No significant association between prenatal SHS and PPD
Ho-Yen et al., 2007	PPD (Yes/No)	Self-reported: assessed by EPDS (PPD >12score EPDS) through structured interview	426	4.90%	6.6 [1.9 - 22]	5.6 (1.2 - 25.6)	Significant association between prenatal ATS and PPD
Tauqeer et al., 2023	PPD (Yes/No)	Self-reported: assessed by Edinburgh Postanal Depression Scale (EPDS) PPD is ≥13 score	1799	17%	1.47 (0.62 to 3.44)		No significant association between prenatal ATS and PPD
Simas et al., 2022	PPD (Yes/No)	Self-reported: assessed by EPDS (PPD≥ 10 score of EPDS)	203	27%		OR=2.96 (95%CI: 1.41 - 6.18)	Significant association between prenatal ATS and PPD
Kang et al., 2022	PPD (Yes/No)	Self-reported: : assessed by Korean version of EPDS (PPD ≥ 10 score of EPDS)	80116	24.30%	7.34 (5.64–9.56)	2.02 (1.44–2.83)	Significant association between prenatal ATS and PPD
Zejnullahu et al., 2021	PPD (Yes/No)	Self-reported: assessed by face to face interview using Albanian translated EPDS. (PPD ≥12 EPDS score)	247	21%	1.088 (0.997–1.187)		No significant association between prenatal ATS and PPD
Lee et al., 2020	PPD (Yes/No)	Self-reported: assessed by EPDS at 3 months after delivery (PPD ≥13 EPDS score).	356 (the original sample size was 1247, after excluding those missing data about either EPDS or life style risk factors, only 356 were included in this analysis)				No significant association between ATS and PPD
Toru et al., 2018	PPD (Yes/No)	Self-reported: assessed by interview using 9-items PHQ9	456	22.40%		5.17, (95%CI: 2.52–10.60)	Significant association between prenatal ATS and PPD
Farr et al., 2014	PPD (Yes/No)	Self-reported: PRAMS questions (Q1: How often since your baby was born do you feel down, depressed or hopeless & Q2: How often since your baby was born do you had little interest or pleasure in doing things). Answers a 5-point Likert scale (“Always”, “Often”, “Sometimes”, “Rarely”, or “Never”). PPD (Yes: “Always”, “Often”/No: “Sometimes”, “Rarely”, or “Never”)	4451	9%		OR= 1.4; 95%CI: 0.7 - 2.7	No significant association between ATS and PPD
Sidebottom et al., 2014	PPD (Yes/No)	Self-reported: assessed by PHQ-9 questions (each question has answers scored 0 - 3) the scores of all questions were then summed (PPD≥ 10)	594	6%		OR: 1.36 (95%CI:0.56 - 3.31)	Significant association between prenatal ATS and PPD
Pooler et al., 2013	PPD (Yes/No)	Self-reported: PRAMS questions (Q1: How often since your baby was born do you feel down, depressed or hopeless & Q2: How often since your baby was born do you had little interest or pleasure in doing things). Answers a 5-point Likert scale (“Always”, “Often”, “Sometimes”, “Rarely”, or “Never”). PPD (Yes: “Always”, “Often”/No: “Sometimes”, “Rarely”, or “Never”)	75,234	Overall prevalence (WIC and non-WIC: 13.8%), prevalence among WIC participants: 19.8%, prevalence among WIC eligible but not participants: 16.3%, prevalence among women not eligible to WIC: 6.8%	Overall OR: 1.65 (95%CI: 1.46 - 1.85)	OR: 1.59 (95%CI: 1.42 - 1.79)	Significant association between prenatal ATS and PPD
Sylvén et al., 2013	PPD (Yes/No)	Self-reported: assessed by structured questionnaire and measured by EPDS. (PPD≥12 EPDS score)	329	11.70%	OR: 1.73 (95%CI: 0.13 - 22.53)	OR: 1.57 (0.08 - 29.12)	No significant association between prenatal ATS and PPD
Skalkidou et al., 2009	PPD (Yes/No)	Self-reported: Assessed by EPDS (the Swedish version) the cutoff point is ≥ 12 EPDS score.	347	This is a case control study		linear regression coefficient: 0.87	No significant association between prenatal ATS and PPD
Kondracki et al., 2024	PPD (Yes/No)	Self-reported: PRAMS questions (Q1: How often since your baby was born do you feel down, depressed or hopeless & Q2: How often since your baby was born do you had little interest or pleasure in doing things). Answers a 5-point Likert scale (“Always”, “Often”, “Sometimes”, “Rarely”, or “Never”). PPD (Yes: “Always”, “Often”/No: “Sometimes”, “Rarely”, or “Never”)	51220	This is a case control study		Low-intensity smokers (1st &2nd Trimester): OR 1.22(95CI%: 1.18 - 1.25)	Significant association between prenatal ATS and PPD
						High-intensity smoker (1st&2nd Trimester): OR=1.21 (95%CI: 1.17 - 1.26)	
						All trimesters (low-intensity): OR=1.17 (95%CI: 1.15 - 1.19)	
						All trimesters (high-intensity): OR=1.165 (95%CI: 1.63 - 1.68)	
						Reduced smoking towards the end of pregnancy: OR=1.13 (95%CI: 1.10 - 1.17)	
						Increased towards the end of pregnancy: OR=0.58 (95%CI: 0.45 - 0.74)	
Alhammadi et al., 2023	PPD (Yes/No)	Assessed by EPDS this scale questions were translated into Arabic and bac translated into English by two independent authors	354	56.20%	0.78		No significant association between prenatal ATS and PPD, and No significant association between prenatal SHS and PPD
Jeon et al., 2024	PPD (Yes/No)	Defined as depression-related diagnostic code “depression-related diagnosis codes was based on Clinical Classification Software (CCS) provided by the Agency for Healthcare Research and Quality (AHRQ).”	61,226	Overall prevalence: 4.04%, (PPD in adults: 3.8%), (PPD in adolescents: 6.08%)	(OR: 1.28 [95% CI: 1.12–1.41] in adults; OR: 1.86 [95% CI: 1.40–2.47] in adolescents)		Significant association between prenatal ATS and PPD
Wiener et al., 2023	PPD (Yes/No)	ICD 10 code	10598		(RR: 1.75 [95% CI: 1.55, 1.9] in unmatched data; RR: 0.68 [95% CI: 0.59, 0.79] matching upon age, race, supervision of high-risk pregnancy, gestational diabetes mellitus, diabetes mellitus, mental/behavioral/neurodevelopmental disorder, person with potential health hazards related to socioeconomic status and psychosocial concerns, preterm (premature) newborn, extreme immaturity of newborn and another low birthweight newborn)	1.75	Significant association between nicotine dependence and PPD
Oliveira et al., 2023	PPD (Yes/No)	Assessed using the Edinburgh Postpartum Depression Scale (EPDS). PPD ≥ 13 EPDS	101	24.80%	3.02 (1.35–8.79)	2.15 (1.51–4.66)	Significant association between prenatal ATS and PPD
Song et al. 2019	PPD (Yes/No)	Assessed by Chines version of EPDS. PPD ≥ 10 EPDS	8842	8.50%	Active smoking 1.00 (95%CI: 0.83–1.18), passive smoking 1.13 (95% CI: 1.02–1.26)	1.43, (95 CI: 1.16–1.77)	Significant association between prenatal SHS and PPD, No significant association between prenatal ATS and PPD
Silva et al., 2021	PPD (Yes/No)	Assessed by EPDS > 10 score	227		1.08 (0.57 - 2.03)		No significant association between prenatal ATS and PPD
Shang et al., 2023	PPD(Yes/No)	Assessed by EPDS	2447	14.80%		(Active smoking: OR=3.86, 95%CI 2.37–6.28) & (Passive smoking: OR = 1.56, 95%CI 2.00–5.71)	Significant association between prenatal SHS and PPD, and significant association between prenatal ATS and PPD
Mora et al., 2009	PPD (Yes/No)	Measured utilizing the Center for Epidemiologic Studies Depression (CES-D) Scale. Cutoff points of ≥16 and ≥23 score indicating significant levels of depressive symptomatology	1735	9%	OR =1.09	OR = 0.92 (0.85 - 1.45)	No significant association between prenatal ATS and PPD

Regarding exposure types, 22 studies assessed ATS only, (Barber and Shenassa 2021; Cui et al. 2020; Farr et al. 2014; Haile et al. 2024; Harrison et al. 2023; Ho-Yen et al. 2007; Jeon et al. 2024; Kang et al. 2022; Kondracki et al. 2024; Moore Simas et al. 2022; Mora et al. 2009; Nunes and Phipps 2013; Pooler et al. 2013; Sidebottom et al. 2014; Silva et al. 2021, 2023; Skalkidou et al. 2009; Sylvén et al. 2013; Tauqeer et al. 2023; Toru et al. 2018; van Lee et al. 2020; Zejnullahu et al. 2021) four assessed both ATS and SHS, (Alhammadi et al. 2023; Dagher and Shenassa 2012; Shang et al. 2023; Song et al. 2019) one assessed SHS only, one examined ENP use, (Choi et al. 2024) and one assessed nicotine dependence (Supplementary Table 1). (Wiener 2023) Overall, 15 studies found significant associations between PEN and PPD, 10 found no association, and four showed associations in specific subgroups only. Of 26 studies that assessed ATS, 13 reported a significant association. Among studies assessing SHS, 3 of 5 reported significant associations. The one ENP study found no significant association, while the one on nicotine dependence reported a positive association.

Study quality scores using the NOS ranged from 3 to 9. Sixteen studies were rated as high quality (scores 7–9), and the remaining 13 as fair quality (scores 3–6). No study was rated poor. Full details are presented in Table 2.

**Table 2:** Quality assessment of the studies included in the systematic review and meta-analysis

Cohort	Representativeness of the exposed cohort	Selection of the non-exposed cohort	Ascertainment of exposure	Demonstration of the outcome of interest was not present at start of study	Comparability of cohorts on the basis of the design or analysis	Assessment of outcome	Was follow-up long enough for outcomes to occur	Adequacy of follow-up of cohorts	Total Score	Study Quality
Dagher et al., 2012	**	*	*	*	*	*	*	-	7	High
Lee et al. 2020	-	-	-	*	*	-	*	-	3	Fair
Sidebottom et al. 2014	*	*	*	**	*	*	*	-	8	High
Wiener et al., 2023	*	*	*	*	*	*	*	-	7	High
Song et al. 2019	*	*	*	*	*	*	*	*	8	High
Mora et al. 2009	*	*	*	*	*	-	*	-	6	Fair
Jeon et al. 2024	*	*	*	*	*	*	*	-	7	High
Case Control Studies	Is the case definition adequate	Representativeness of the cases	Selection of the controls	Definition of the controls	Comparability of cases and controls on the basis of the design or analysis	Ascertainment of the exposure	Same methods of ascertainment for cases and controls	Non-response rate	Total Score	Study Quality
Skalkidou et al., 2009	-	*	*	*	*	*	*		6	Fair
Kondracki et al. 2024	-	*	-	-	*	-	*		3	Fair
Silva et al. 2021	*	*	*	*	*	*	*	*	8	High
Cross sectional Studies	Representativeness of the cases	Sample Size	Non-Response rate	Ascertainment of the screening/surveillance tool	The potential confounders were investigated by subgroups analysis or multivariable analysis	Assessment of the outcome	Statistical test		Total Score	Study Quality
Barber et al., 2021	1	1	0	1	1	1	1		6	Fair
Haile et al. 2024	1	1	0	1	1	1	1		6	Fair
Nunes., 2013	1	1	0	1	1	1	1		6	Fair
Harrison et al. 2023	1	1	0	2	1	1	1		7	High
Khan et al. 2015	1	1	0	1	1	1	1		6	Fair
Choi et al.., 2024	1	1	0	1	1	1	1		6	Fair
Cui et al. 2020	1	1	1	2	1	2	1		9	High
Ho-Yen et al., 2007	1	1	0	2	1	1	1		7	High
Tauqeer et al. 2023	0	0	0	2	1	1	1		5	Fair
Simas et al., 2022	0	0	0	2	1	2	1		6	Fair
Kang et al. 2022	1	1	0	2	1	1	1		7	High
Zejnullahu et al. 2021	1	0	0	1	1	1	1		5	Fair
Toru et al. 2018	1	1	1	2	1	1	1		8	High
Farr et al. 2014	1	1	1	1	1	1	1		7	High
Pooler et al. 2013	1	1	0	1	1	1	1		6	Fair
Sylvén et al. 2013	1	1	0	2	1	2	1		8	High
Alhammadi., 2023	1	0	1	2	0	1	0		5	Fair
Oliveira et al., 2023	1	0	0	2	1	1	1		6	Fair
Shang et al., 2023	1	0	0	2	1	1	1		6	Fair

### Meta-analysis

Out of the 29 studies included in the systematic review, 26 met the criteria for inclusion in the meta-analysis. Some studies reported stratified results, yielding 33 effect size estimates and covering a total of 642,959 participants.

### Prevalence of postpartum depression

Among the 26 studies, 16 provided individual-level data suitable for estimating the PP of PPD. The overall PP was 0.15, 95%CI[0.12–0.17], with substantial heterogeneity observed across studies (I²=99.8%,*p* < 0.001). When PP stratified by PEN status, PPD was significantly higher among women with PEN compared to unexposed women (PEN-PP = 0.23, 95%CI[0.19–0.27]; *p* < 0.001 vs. NoPEN-PP = 0.14, 95%CI[0.13–0.17]; *p* < 0.001), Supplementary Table 2. There was significant heterogeneity in PPD prevalence between studies. Therefore, subgroup PP were calculated by type of PEN (ATS, SHS, or ENP), study site, and method of PPD ascertainment.

Subgroup analysis revealed essential differences in PPD prevalence based on type of PEN, study site, and method of PPD ascertainment. Studies assessing ATS showed the highest prevalence (PP = 0.25), while SHS and ENP use had lower PP estimates of (0.10) and (0.19), respectively. Geographically, South America had the highest PP (0.28), followed by the U.S. (0.11) and Asian countries (0.9). Regarding the PPD ascertainment method, higher PP was observed in studies using EPDS or PHQ-9 compared to those relying on PRAMS questions or clinical diagnostic tools. The variability in PPD prevalence across studies using EPDS likely reflects differences in cutoff thresholds, translated EPDS versions, timing and method of data collection.

### Association between PEN and PPD

**Fig. 2:**
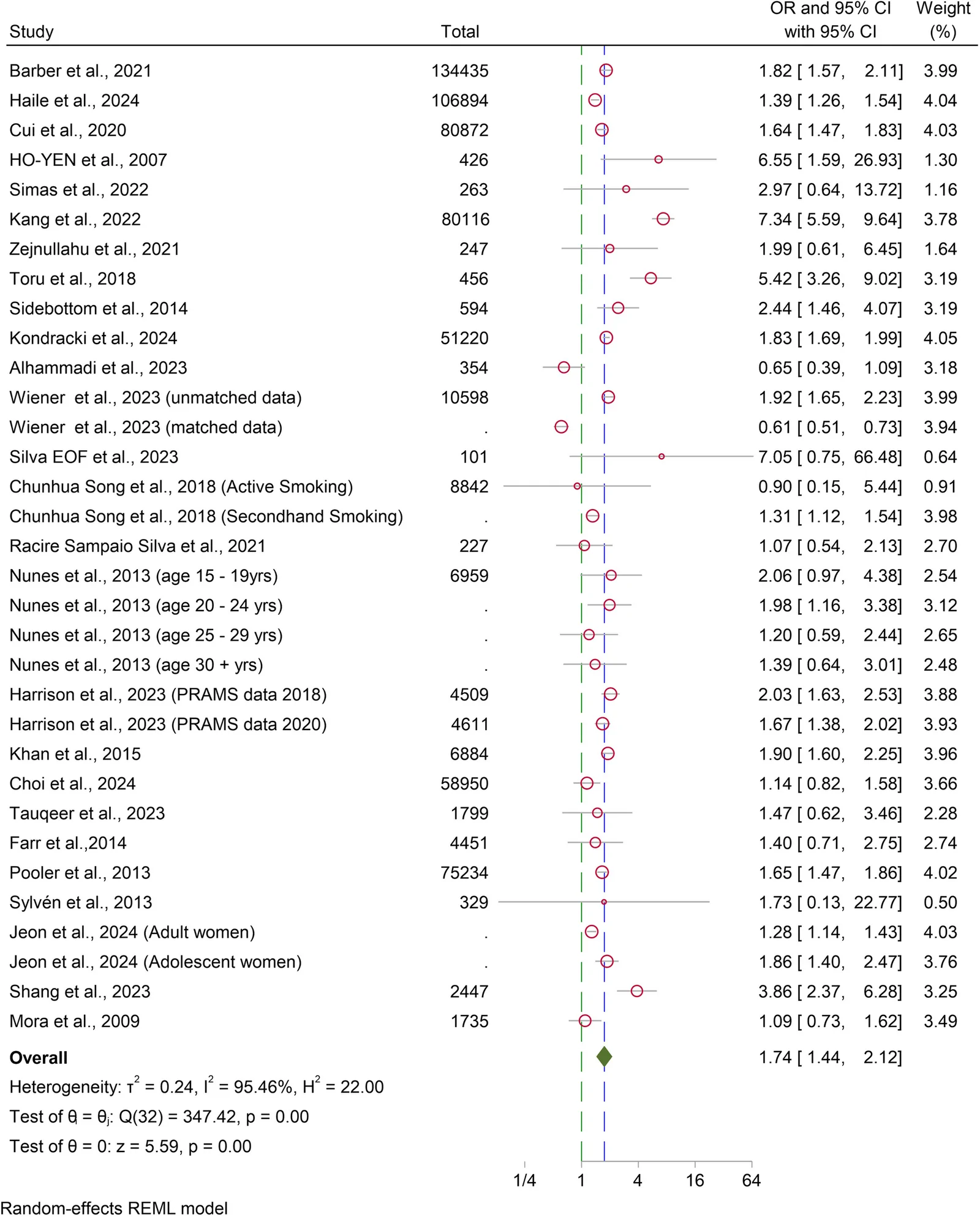
Forest plots of all studies included in the meta-analysis

**Fig. 3:**
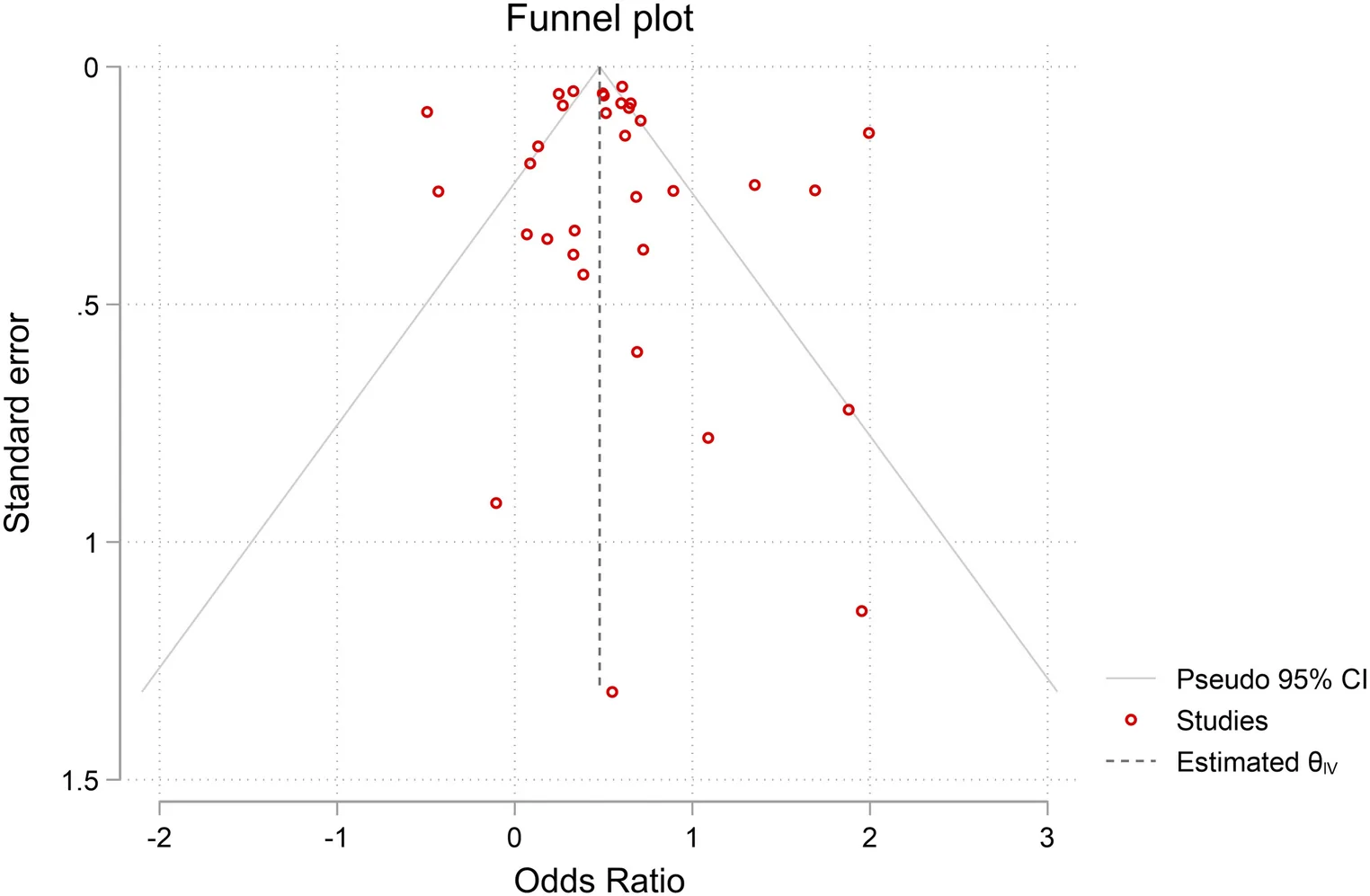
Funnel plot to assess for publication bias

The meta-analysis of 33 odds ratios showed that PEN was significantly associated with increased odds of PPD. The overall pooled-OR was (1.74, 95%CI[1.44–2.12]; Z = 5.59,*p* < 0.001), indicating 74% higher odds of PPD among women with PEN compared unexposed women **(**Fig. 2**).** While visual inspection of the funnel plot revealed some asymmetry **(**Fig. 3**)**, Egger’s tests showed no significant evidence of publication bias (*p* = 0.343), suggesting the asymmetry was more likely due to between-study heterogeneity.

Subgroup analysis by type of exposure showed that ATS was associated with significantly elevated odds of PPD (pooled-OR = 1.96, 95%CI[1.59–2.41]; Z = 6.46,*p* < 0.001). In contrast, SHS (pooled-OR = 1.22, 95%CI[0.69–2.18]; Z = 0.69,*p* = 0.49) and ENP (pooled-OR = 1.14, 95%CI[0.82–1.58]; Z = 0.78,*p* = 0.43) exposures were not significantly associated with PPD. One study examining nicotine dependence found inconsistent findings, with a matched analysis showing a protective association (OR = 0.61, 95%CI[0.51–0.73]; Z=−5.21,*p* < 0.001) and an unmatched analysis indicating increased risk (OR = 1.92, 95%CI [1.65–2.23]; Z = 8.49,*p* < 0.001), Supplementary Fig. 1.a.

When stratified by method of PPD ascertainment, all groups demonstrated elevated odds of PPD among women with PEN. Studies using EPDS had the highest pooled-OR (2.01, 95%CI[1.36–2.97]; Z = 3.52,*p* < 0.001), followed by studies using other clinical tools (1.66, 95%CI[1.01–2.72]; Z = 1.99,*p* < 0.001), and PRAMS-based studies (1.64, 95%CI[1.47–1.83]; Z = 8.77,*p* < 0.001), Supplementary Fig. 1.b. Heterogeneity was highest in studies using EPDS that could be due to differences in scale versions, cutoff values, and languages. Also, high heterogeneity was observed among studies that grouped diverse diagnostic tools together. The funnel plot is symmetrical for both EPDS and PRAMS groups but not for the third group. However, Egger’s tests showed no significant evidence of publication bias, Supplementary Fig. 2.a.

Stratification by study site showed that both U.S.-based (pooled-OR = 1.51, 95%CI[1.28–1.79]; Z = 4.79,*p* < 0.001) and non-U.S. studies (pooled-OR = 2.15, 95%CI[1.44–3.22]; Z = 3.73,*p* < 0.001) reported significantly higher odds of PPD among women with PEN, Supplementary Fig. 1.c, although heterogeneity remained high in both group, funnel plots and Egger’s test showed no evidence of publication bias, Supplementary Fig. 2.b. This may be attributable to variations in population characteristics and methodological differences.

By study design, cross-sectional and case-control studies (combined) yielded a pooled-OR of (1.92, 95%CI[1.53–2.42]; Z = 5.57,*p* < 0.001), while cohort studies showed a pooled-OR of (1.34, 95%CI[0.97–1.86]; Z = 1.79,*p* = 0.071), which was not statistically significant. Both groups exhibited substantial heterogeneity, possibly due to variation in sample size, study populations, and PPD ascertainment methods, Supplementary Fig. 1.d. The funnel plot and Egger’s tests showed no significant evidence of publication bias or small study effect, Supplementary Fig. 2.c.

Based on NOS quality assessment, both high and fair quality studies showed significant associations between PEN and PPD; High-quality (pooled-OR = 1.89, 95%CI[1.35–2.65]; Z = 3.68,*p* < 0.001) and Fair-quality (pooled-OR = 1.60, 95%CI[1.33–1.93]; Z = 4.98,*p* < 0.001) with substantial heterogeneity across both, Supplementary Fig. 1.e, but no evidence of publication bias or small study effect, Supplementary Fig. 2.d. 

**Fig. 4:**
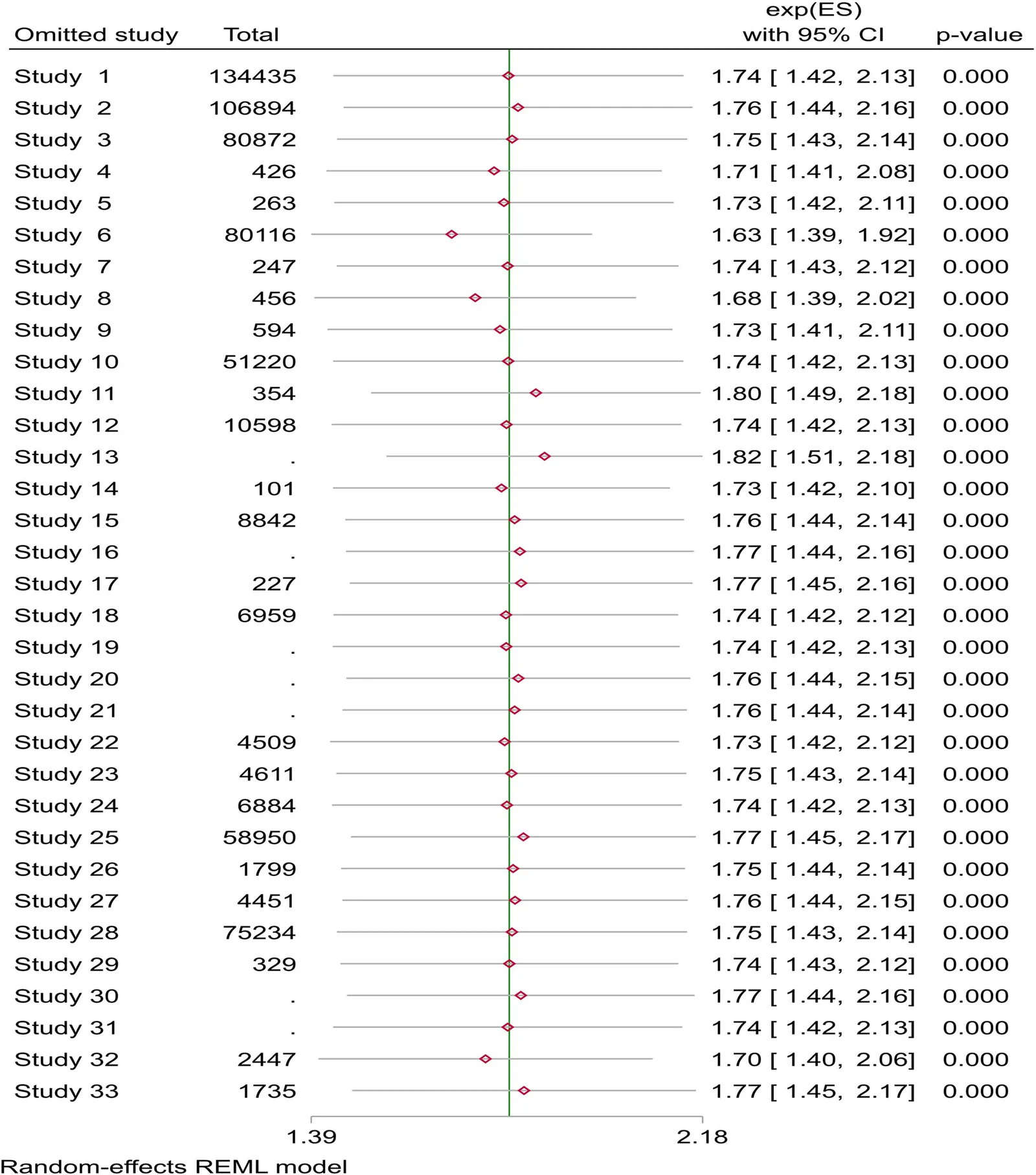
Forest plot of leave one out analysis

Meta-regression analysis did not identify any study-level variables—including study site, design, exposure type, or quality—as significant contributors to heterogeneity (Table 3). Sensitivity analyses using leave-one-out methods showed no influential studies that altered the pooled estimate meaningfully (Supplementary Table 4, Fig. 4). A trim-and-fill analysis introduced three imputed studies and resulted in an adjusted pooled-OR of (1.57, 95%CI[1.52–1.63]), which was nearly identical to the unadjusted pooled-OR (1.61, 95%CI[1.56–1.67]), indicating minimal influence from potential publication bias (Supplementary Table 5).

**Table 3:** Meta Regression showing the adjusted pooled odds ratio and 95% confidence interval of the 26 studies included in the meta-analysis

Meta-regression	*N*	Exp (ES): OR	95% Confidence Interval]	Z	*P*-value
Tool of PPD Ascertainment					
EPDS	15	1	Reference		
PRAMS	11	0.53	[0.09 - 3.28]	−0.68	0.498
Other method	7	0.89	[0.11 - 7.15]	−0.11	0.913
Study Design					
Cross-sectional	23	1	Reference		
Cohort	8	0.42	[0.16 - 1.15]	−1.69	0.092
Case-control	2	0.91	[0.36 - 2.28]	−0.21	0.837
Quality of the study					
Fair quality	17	1	Reference		
High quality	16	1.27	[0.74 - 2.17]	0.88	0.379
Study Site					
North America	18	1	Reference		
Europe	5	0.51	[0.07 - 3.40]	−0.7	0.485
Asia	7	0.76	[0.12 - 4.99]	−0.28	0.78
South America	2	0.45	[0.05 - 4.25]	−0.69	0.488
Africa	1	1.61	[0.35 - 7.35]	0.62	0.536

## Discussion

This study was conducted to address a critical gap in literature by systematically examining the overall impact of PEN, including ATS, SHS, and ENPs, on the risk of PPD. The meta-analysis of 26 studies provided a consistent evidence that such exposure is associated with increased odds of PPD. The pooled prevalence of PPD was (15%), and women with PEN had significantly higher odds of developing PPD compared to those unexposed. Although there was considerable heterogeneity among studies, the association remained robust across sensitivity analyses and was not likely driven by publication bias.

### Pooled Prevalence of PPD

The estimated PP of PPD in our meta-analysis is (15%). This is consistent with findings from previous systematic reviews. These include a global review covering 80 countries (17.22%), (Wang et al. 2021) a review focusing exclusively on women without a prior history of depression (17%), (Shorey et al. 2018) and another review that included studies up to 2020 (14%). (Liu et al. 2022) However, our estimate exceeds that of a more recent review (12%) that was restricted only to studies that employed diagnostic tools to define PPD. (Bai et al. 2023) In our analysis, only two studies used diagnostic tools, yielding PP of (11%), closely aligned with the lower estimate of the above mentioned review. (Bai et al. 2023) The higher overall PP in our findings is likely attributed to the predominance of studies using self-reported screening tools, which are known to overestimate PPD rates compared to diagnostic assessments. (Upadhyay et al. 2017)

In contrast, our PP is lower than estimates reported in region-specific or population-focused reviews. For example, higher rates have been documented in Sub-Saharan Africa (22.1%) (Nweke et al. 2024) Uganda (29%), (Kabunga et al. 2024) India (22%), (Upadhyay et al. 2017) Ethiopia (22.08%), (Zeleke et al. 2021) and (21.9%), (Necho et al. 2020) Italy (27.5%), (Camoni et al. 2023) and a meta-analysis included 18 low and lower-middle-income countries (18.6%). (Fisher et al. 2012) Our estimate is also notably lower than that of a recent meta-analysis (31%) which included only studies conducted during the COVID-19 pandemic. (Vilarim et al. 2024) Among the six studies in our review from Asian countries (China, Japan, South Korea, Singapore, and Nepal) the PP was (9%), (Cui et al. 2020; Ho-Yen et al. 2007; Kang et al. 2022; Shang et al. 2023; Song et al. 2019; van Lee et al. 2020) which is significantly lower than both our overall estimate and previous meta-analyses estimates from Asian countries.

Several factors may explain the observed discrepancies in PPD prevalence across studies. The countries included in our meta-analysis differ from those in earlier reviews and may have stronger healthcare systems and more favorable social determinants of health, contributing to lower PPD rates. Additionally, the recency of the studies may reflect a shift in PPD trends over time. Another key contributor to the variability is the methodology used to assess PPD. Prior research has shown that self-reported screening tools (such as the EPDS) tend to yield higher estimates compared to clinical diagnostic instruments. (Bai et al. 2023; Upadhyay et al. 2017) Consistent with this, our analysis found a PP of (17%) in studies using screening tools, compared to (11%) in those utilized diagnostic tools.

The subgroup analysis revealed a PP of (25%) among women who reported prenatal ATS, compared to (13%) among non-smokers. These rates are substantially higher than those reported in a 2017 meta-analysis, which found PP of (3%) and (1.3%) among smokers and non-smokers, respectively. (Chen et al. 2019) This disparity may reflect differences in inclusion criteria and the more recent timeframe of the studies included in our meta-analysis, potentially indicating a rising trend in PPD that warrants further investigation. In contrast, the PP among women exposed to SHS during pregnancy was (10%) compared to (9%) among unexposed women, suggesting a minimal difference. However, these findings should be interpreted with caution, as only three studies contributed to this analysis, all relying on self-reported SHS exposure without objective measures. This reliance increases the likelihood of exposure misclassification, which may attenuate the association toward the null.

### The association between prenatal exposure to nicotine and PPD

This meta-analysis found that PEN was associated with a ~ 70% increase in the odds of PPD compared to no exposure. However, the heterogeneity among included studies was high (I² = 95.46%), likely reflecting true variability rather than publication bias. Possible contributors include differences in study populations (across multiple continents), study design, data sources (e.g., primary data vs. medical records), quality of studies, and variability in PPD ascertainment tools. The overall pooled estimate of the association between PEN and PPD must be interpreted cautiously due to substantial heterogeneity between studies. Therefore, the strata-specific pooled estimates by type of PEN are considered the principal findings for this meta-analysis in particular the robust association observed for ATS.

Women who reported prenatal ATS had nearly twice the odds of developing PPD compared to non-smokers (pooled-OR = 1.96). This finding aligns with a previous meta-analysis that reported a similar association between prenatal tobacco use and PPD (pooled-OR = 2.325). (Chen et al. 2019) Our findings are also consistent with a more recently published review that also showed a positive correlation between smoking during pregnancy and risk of PPD with a dose-response relationship. (Knysak et al. 2025) While Knysak et al., review contributed important and most updated insight, there are methodological and conceptual distinctions underscores the novelty and added value of our study. First, Knysaks’ review was conducted as a narrative review of 10 studies compared to ours that is a systematic review and meta-analysis of 29 studies. Second, we had a more comprehensive search covering 5 databases across 25 years span. Finally, our systematic review and meta-analysis expanded the scope to include overall prenatal exposure to nicotine incorporating evidence about ATS, SHS, and ENPs use as well as separate analysis for each type of PEN. SHS and ENPs use were not addressed in the previous review.

It is worth noting that despite the strong pooled estimate of ATS with PPD, individual studies showed inconsistent results, which may reflect underreporting of tobacco use during pregnancy, particularly in studies relying on self-reported data. (Dietz et al. 2011; Ford et al. 1997; Owen and McNeill 2001; Wong and Koren 2001) Social desirability bias may lead women to deny smoking, resulting in misclassification of smokers as non-smokers and producing nondifferential misclassification of exposure. Additionally, most of the data collected retrospectively, therefore estimates of exposure are prone to recall bias leading also to a non-differential misclassification. Both, in turn, likely attenuates the observed association toward the null, suggesting that the true effect of prenatal ATS on PPD may be underestimated.

In this meta-analysis, no significant association was found between prenatal SHS exposure and PPD (pooled-OR = 1.22). Only three studies assessed this relationship. While two reported significant associations, the pooled result was non-significant. These findings contrast with a 2018 meta-analysis that reported increased PPD risk with SHS exposure. (Suzuki et al. 2019) The inconsistency may stem from differences in exposure definitions and limited sample sizes. For instance, one excluded study assessed SHS solely as partner smoking in the mother’s presence, likely underestimating true exposure. (Eiden et al. 2011) Additionally, most studies relied on self-reported SHS exposure without objective biomarkers, increasing the risk of nondifferential misclassification and attenuating observed associations. (Artzi-Medvedik et al. 2022) This may explain the similar PPD prevalence observed between SHS-exposed (10%) and non-exposed (9%) women in our meta-analysis. Future studies should adopt standardized SHS definitions and objective tools to ascertain SHS(such as cotinine testing) to improve accuracy. Due to the limited number of studies and the large heterogeneity, definitive conclusions about SHS and PPD cannot yet be drawn, and findings about this association from this meta-analysis should be interpreted cautiously.

Despite the increasing trend of ENP use and the tendency of women who used to smoke tobacco to switch to ENPs during pregnancy, we found only one study assessed the association between the use of ENP during pregnancy and PPD. Therefore, we could not provide a pooled estimate for the association between prenatal ENP and PPD, and no concrete conclusion could be reached about this association from our meta-analysis. Subgroup analysis by type of nicotine exposure provided solid conclusion about ATS, however, for SHS and ENPs our findings could be framed as preliminary estimates for future meta-analyses.

Subgroup analysis by study design revealed that cohort studies reported lower and non-significant ORs compared to cross-sectional or case-control studies. This could be inherent to the study design itself, as previous research has shown that non-cohort studies tend to overestimate associations. This also may reflect inherent strengths of the cohort design, which minimizes recall and observer bias by assessing exposure prospectively. Additionally, temporality can be established from studies that adopted cohort design. In contrast, retrospective designs measure both exposure and outcome simultaneously, increasing susceptibility to recall bias and differential misclassification. (Jurek et al. 2005) For example, if assessors are aware of the study hypothesis, they may overemphasize exposure in women with depressive symptoms, further inflating associations. However, cohort design is prone to selection bias mainly by loss to follow-up which might overestimate or underestimate the association. Additionally, the majority of the cohort studies included in this meta-analysis have relatively smaller sample sizes (~ 300–700) compared to the other designs; the significance of the association could be lost due to lack of power, particularly if loss to follow-up was high.

Considerable variation in PPD prevalence was observed across studies, likely driven by differences in assessment tools (e.g., EPDS, PRAMS, PHQ-9), cut-off points, and validation. Lower EPDS thresholds generally yield higher prevalence estimates, though no consistent pattern emerged. Language-adapted versions may lack standardized validation. Additional heterogeneity stemmed from differences in populations, sample sizes, and sampling strategies. Primary data studies often lacked transparent recruitment, raising selection bias concerns, while large surveys like PRAMS used probabilistic sampling but had low response rates (e.g., 61%), potentially biasing estimates. Studies using EPDS reported higher odds ratios for the PEN–PPD association, possibly due to greater sensitivity and reduced measurement error. Further research is needed to validate and compare PPD tools, as improving standardization and internal validity is essential for enhancing future research reliability and comparability.

### Limitations

In this meta-analysis there are several limitations, first, the lack of standardized tools for exposure and outcome ascertainment leads to non-differential misclassification that attenuates the association towards the null in the included studies and introduces huge heterogeneity for the pooled estimate in this meta-analysis. All included studies relied on self-reporting for assessing PEN, which is prone to recall and reporting bias, which leads to non-differential misclassification and attenuates the association towards the null. On the Other hand, most of the studies relied on screening tools (e.g., EPDS, PRAMS questions, PHQ9) to assess for PPD, and these tools tend to overestimate PPD prevalence compared to diagnostic interviews hence inflating the estimated Odds Ratios. Furthermore, these tools vary widely, using various scales, and within the same tools, different versions or cutoff points were used to define PPD, leading to huge heterogeneity between the studies, with some studies might overestimate the associations and others suffer non-differential misclassification, which attenuates the association towards the null and hence affects the pooled estimate. Second, comparison groups were defined as women who reported no smoking or ENP use during pregnancy; however, these groups may have included women who quit upon pregnancy recognition, potentially introducing nondifferential misclassification and weakening observed associations. Most studies used routine data that did not distinguish between never-smokers and those who quit during pregnancy. Only a few explicitly defined comparison groups as never-smokers. Additionally, some studies categorized smoking under broader labels like “substance use,” which may obscure the specific effect of tobacco. One such study in our review may have estimated odds reflecting multiple substances rather than tobacco alone, possibly biasing results. Nonetheless, leave-one-out sensitivity analysis indicated that no single study significantly affected the pooled estimates, suggesting overall findings remained robust. Another limitation is that the majority of the included studies are cross-sectional, which has several limitations that hinder causal inference. Compared to cohort design, cross-sectional cannot establish temporality, prone to reporting and recall bias which may overestimate or underestimate the association particularly if the data collector or the participants are aware about the hypothesized association (however, most of those studies used routinely collected data that was not collected for the proposed association between PEN and PPD, therefore unlikely to lead to differential misclassification), albeit, recall bias in these studies can lead to non-differential misclassification and attenuate the association towards the null. Furthermore, databases such as Web of Science were not included in the search strategy for this review, which might lead to missing valuable studies that assessed for this association, however, we expanded our search to the most common databases which cover most of the literature and we searched manually the references of the references to make sure that our search covers all relevant studies. Moreover, the confounding effect by prior mood disorder such as depression or anxiety status could not be ruled out. This is because primary studies included in the analysis varied in whether adjusted for previous depression or anxiety, and our meta-analysis did not stratify the pooled estimate by adjustment status for prior mood disorders. This limitation raises the possibility that the observed associations between PEN and PPD might be overestimated due to underlying vulnerability to mood disorders, which could be associated with both continued smoking during pregnancy and PPD—rather than reflecting a direct causal effect of PEN itself. Finally, we included studies between January 2000 and September 2024, there could be studies published before or after the search dates for this review, therefore inference from our finding should be cautiously interpreted while taking into account this time frame.

## Conclusion

Our meta-analysis showed a significant association between prenatal exposure to nicotine (PEN) and increased odds of postpartum depression (PPD), especially for active tobacco smoking (ATS). The evidence for secondhand smoke (SHS) was inconclusive due to limited studies, and only one study assessed electronic nicotine product (ENP) use. These findings underscore the need for more prospective studies using objective exposure measures and standardized tools to ascertain both PEN and PPD.

## Supplementary Information

Below is the link to the electronic supplementary material.

Supplementary Material 1 Supplementary Fig. ‎1.a: Forest plot by type of nicotine use

Supplementary Material 2 Supplementary Fig. 1.b: Forest plot by assessment method of PPD

Supplementary Material 3 Supplementary Fig. ‎1.c: Forest plot by study site

Supplementary Material 4 Supplementary Fig. ‎1.d: Forest plot by study design

Supplementary Material 5 Supplementary Fig. 1.e: Forest plot by quality of the study

Supplementary Material 6 Supplementary Fig. 2.a: Funnel plots for the Odds Ratio by the assessment method of PPD

Supplementary Material 7 Supplementary Fig. 2.b: Funnel plots for the Odds Ratio by study site

Supplementary Material 8 Supplementary Fig. 2.c: Funnel plots for the Odds Ratio by study design

Supplementary Material 9 Supplementary Fig. 2.d: Funnel plots for the Odds Ratio by quality of the study

Supplementary Material 10

Supplementary Material 11

Supplementary Material 12

Supplementary Material 13

Supplementary Material 14

## Data Availability

No datasets were generated or analysed during the current study.
