# Supplementary figures and images for: Prenatal exposure to nicotine and postpartum depression: a systematic review and meta-analysis
Source: Arch Womens Ment Health. 2026 Jul 1;29(4):102. doi: 10.1007/s00737-026-01739-6 (PMC13323114; doi:10.1007/s00737-026-01739-6)

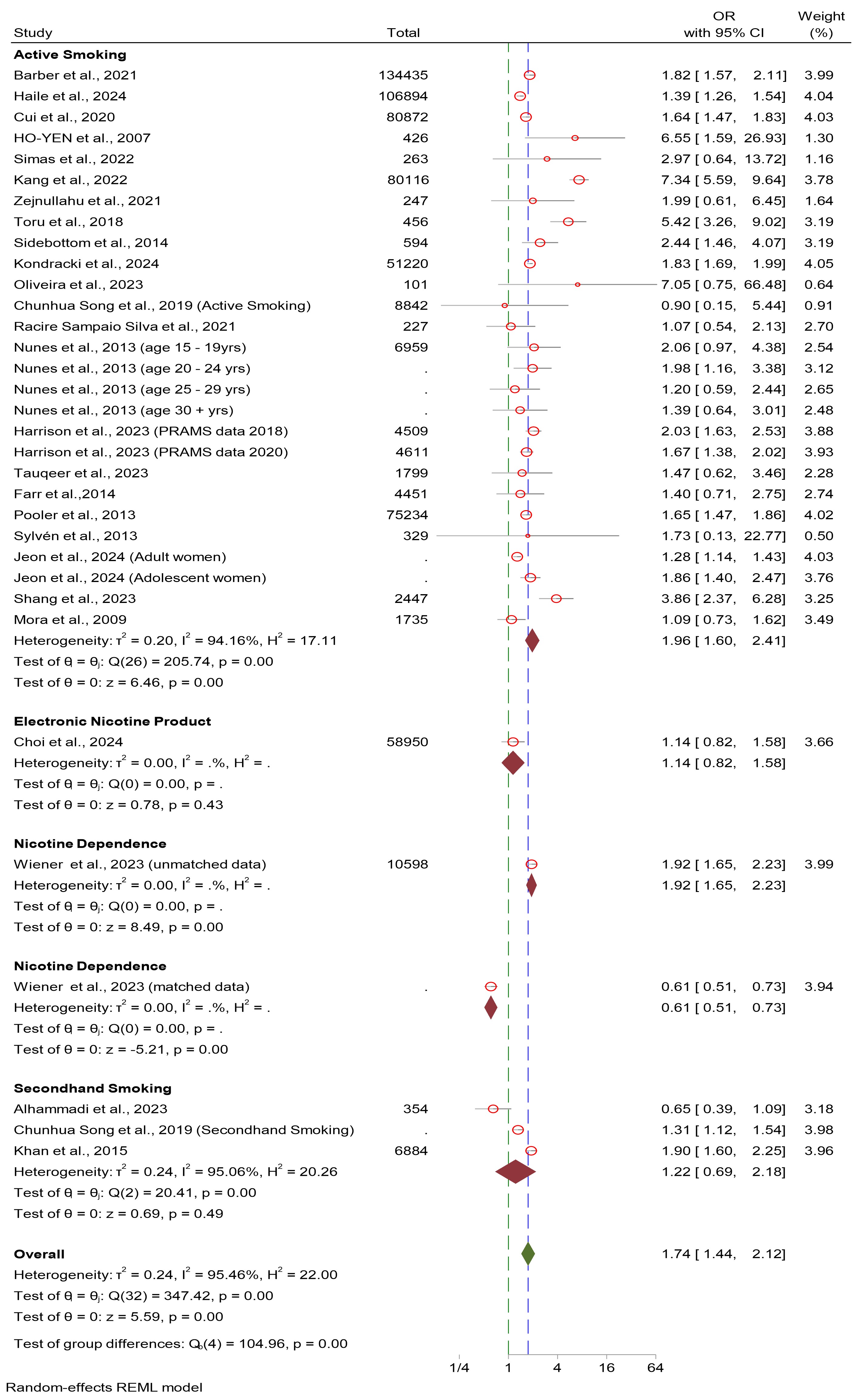

Supplement: Supplementary file 1 — Supplementary Material 1 Supplementary Fig. ‎1.a: Forest plot by type of nicotine use [file 737_2026_1739_MOESM1_ESM.jpg]

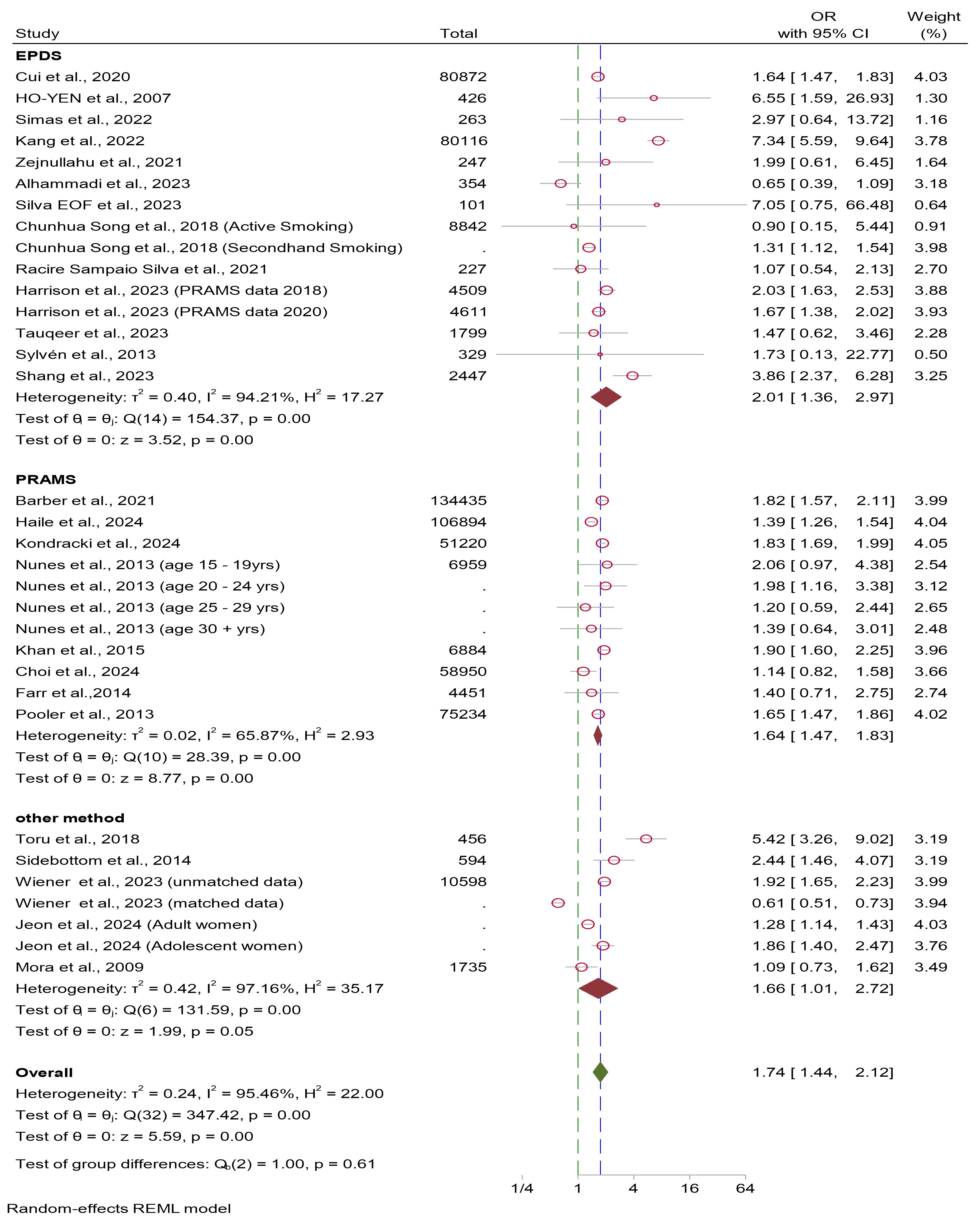

Supplement: Supplementary file 2 — Supplementary Material 2 Supplementary Fig. 1.b: Forest plot by assessment method of PPD [file 737_2026_1739_MOESM2_ESM.jpg]

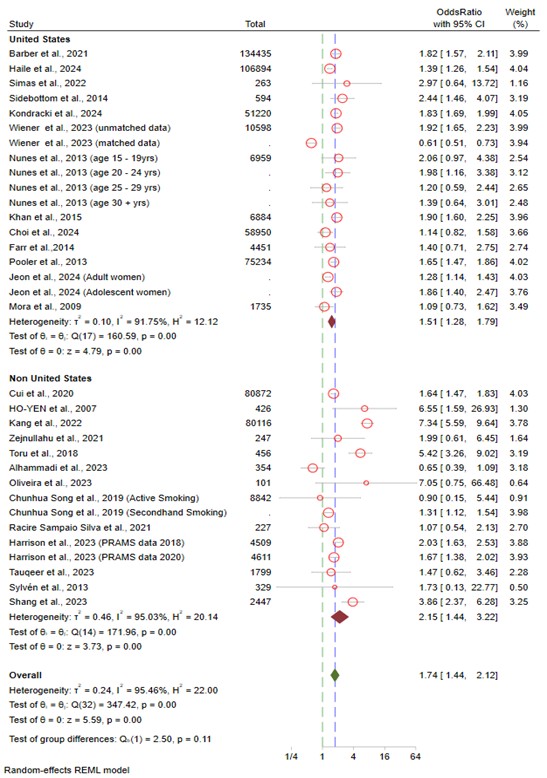

Supplement: Supplementary file 3 — Supplementary Material 3 Supplementary Fig. ‎1.c: Forest plot by study site [file 737_2026_1739_MOESM3_ESM.jpg]

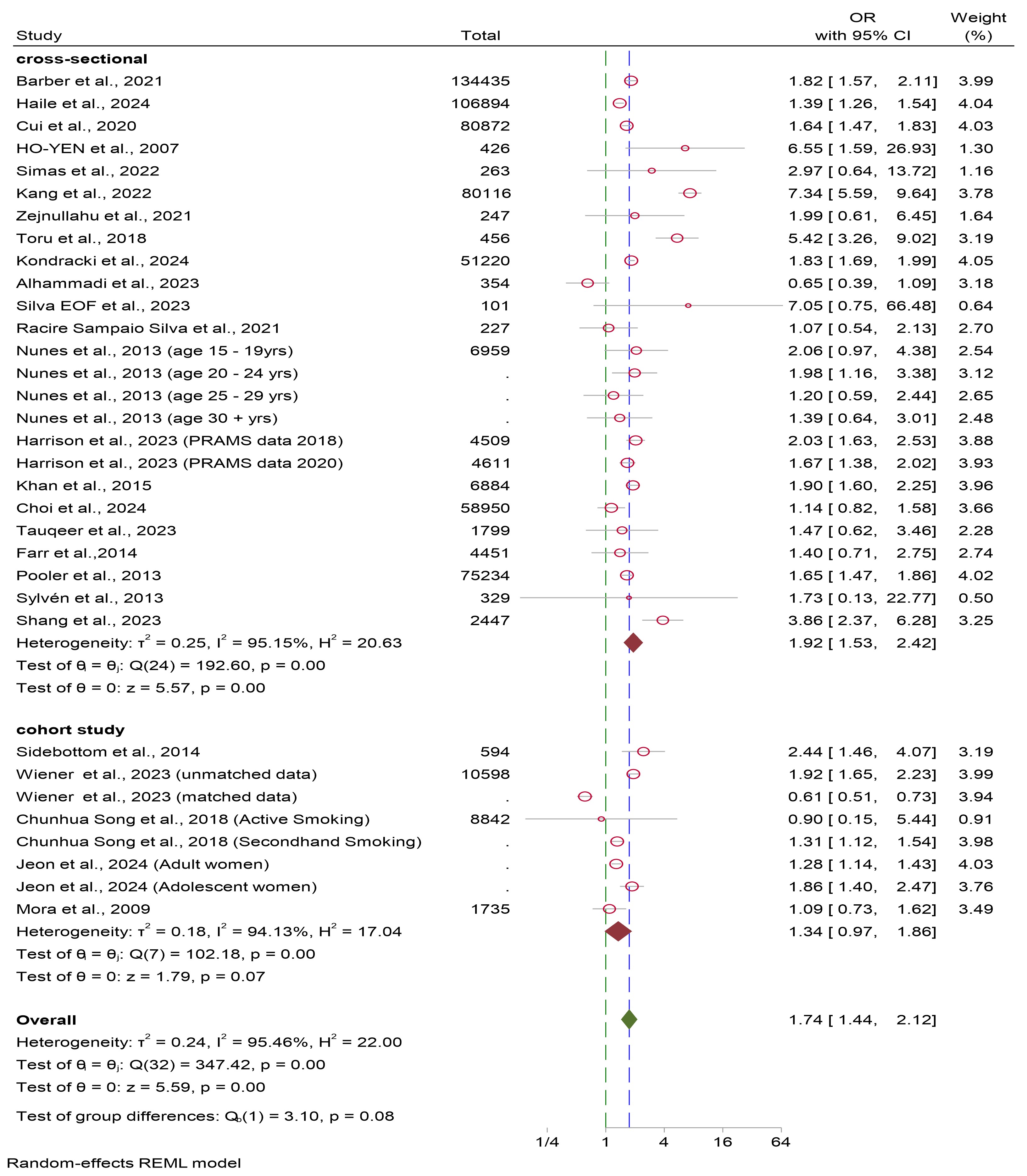

Supplement: Supplementary file 4 — Supplementary Material 4 Supplementary Fig. ‎1.d: Forest plot by study design [file 737_2026_1739_MOESM4_ESM.jpg]

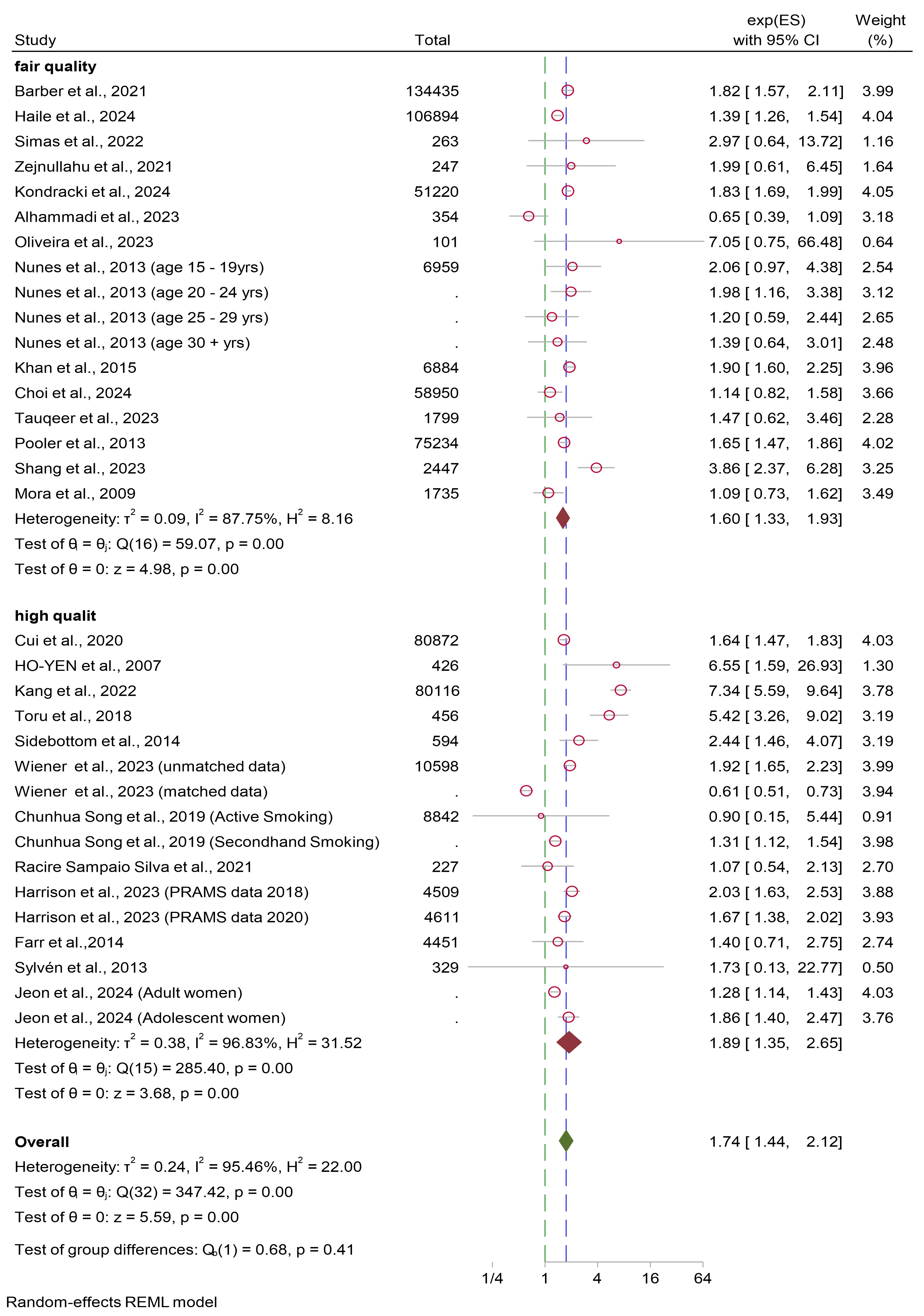

Supplement: Supplementary file 5 — Supplementary Material 5 Supplementary Fig. 1.e: Forest plot by quality of the study [file 737_2026_1739_MOESM5_ESM.jpg]

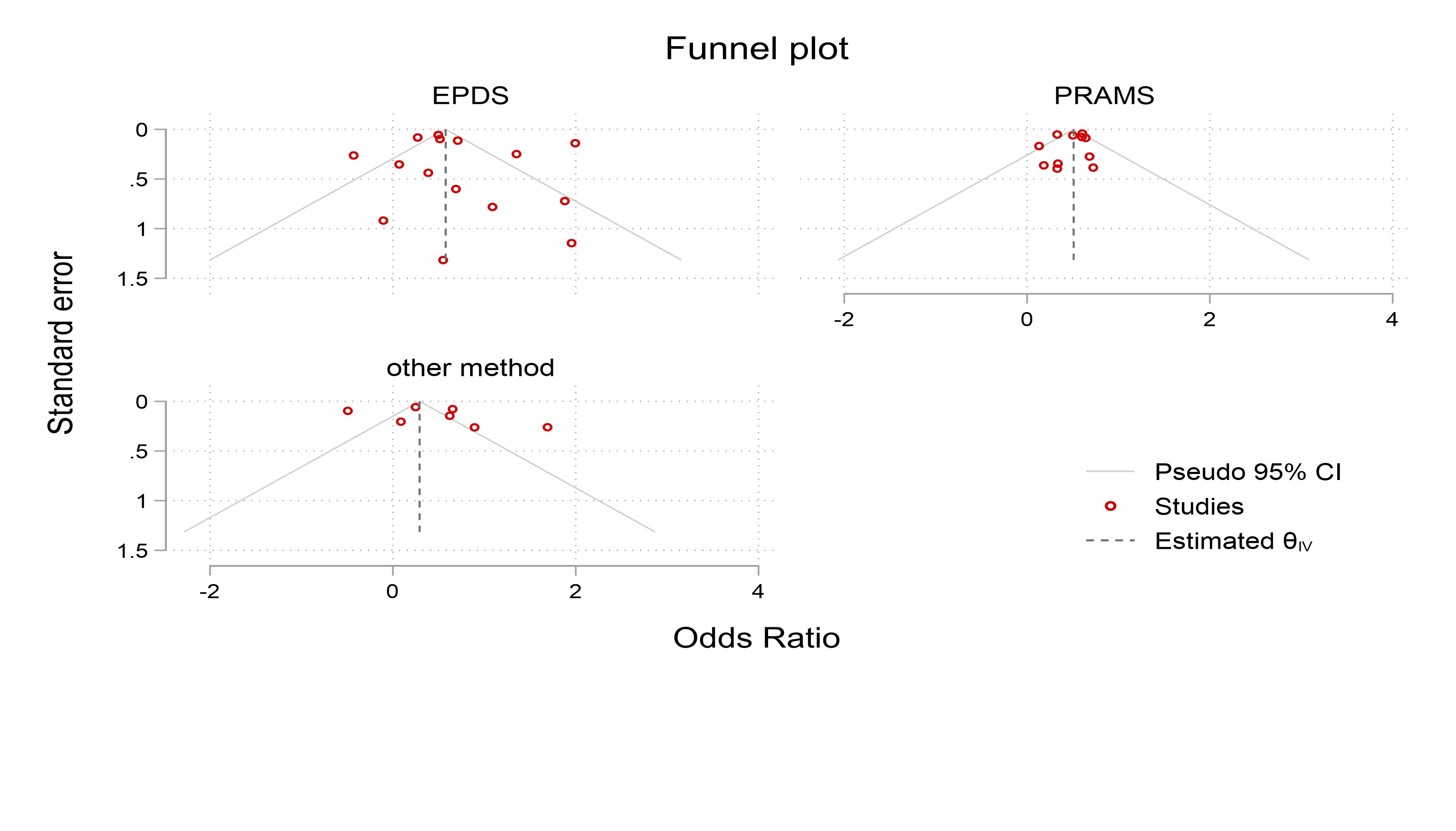

Supplement: Supplementary file 6 — Supplementary Material 6 Supplementary Fig. 2.a: Funnel plots for the Odds Ratio by the assessment method of PPD [file 737_2026_1739_MOESM6_ESM.jpg]

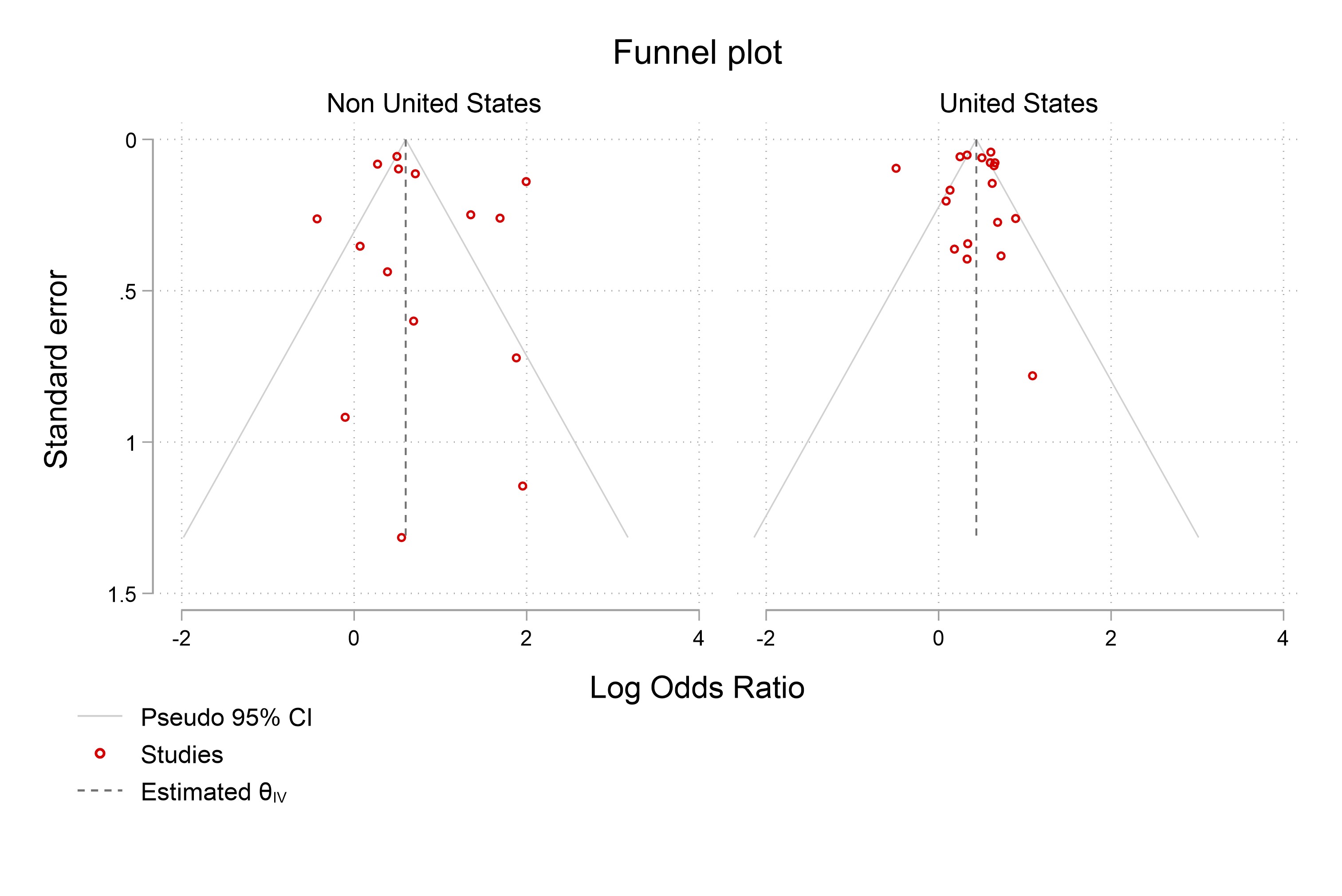

Supplement: Supplementary file 7 — Supplementary Material 7 Supplementary Fig. 2.b: Funnel plots for the Odds Ratio by study site [file 737_2026_1739_MOESM7_ESM.jpg]

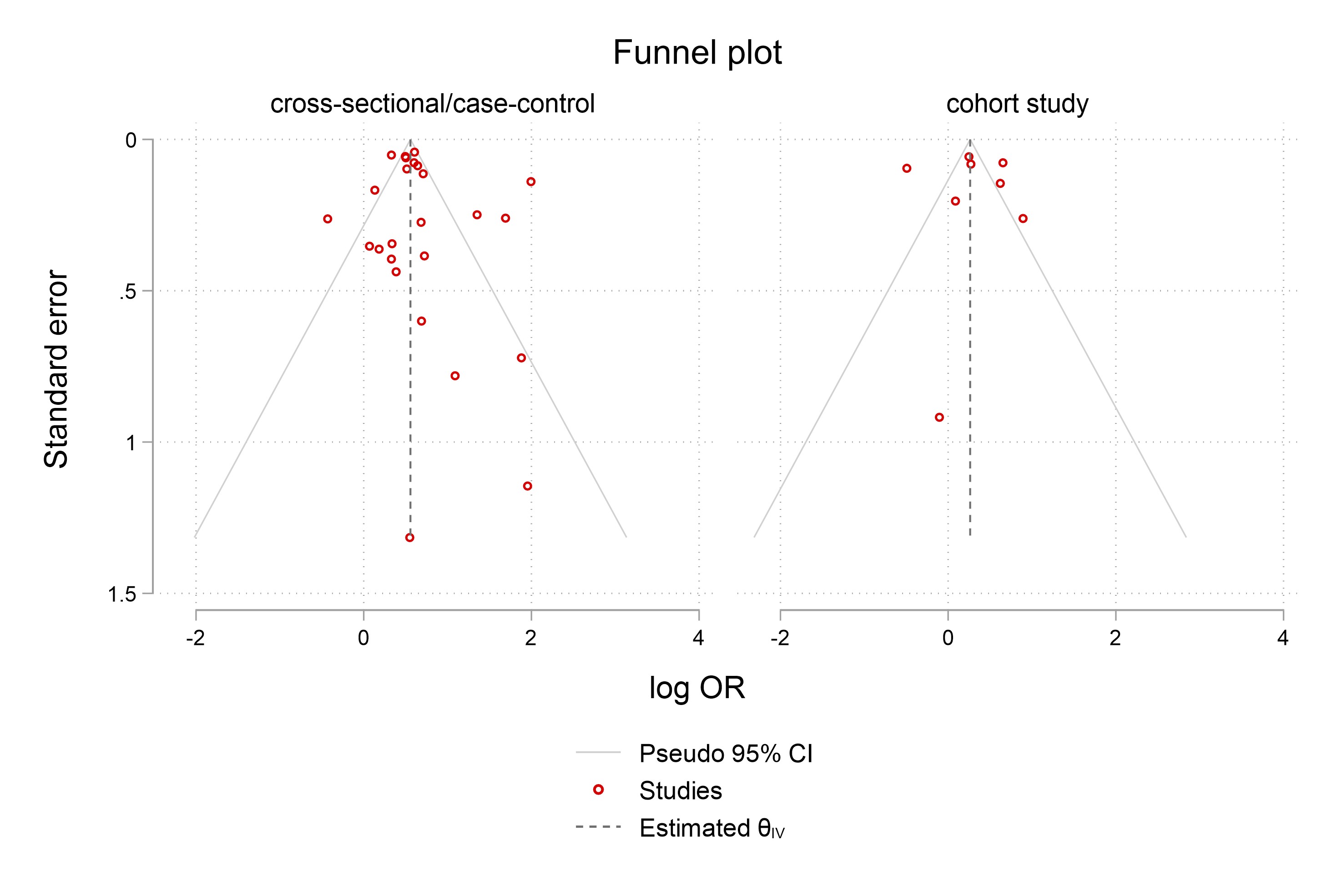

Supplement: Supplementary file 8 — Supplementary Material 8 Supplementary Fig. 2.c: Funnel plots for the Odds Ratio by study design [file 737_2026_1739_MOESM8_ESM.jpg]

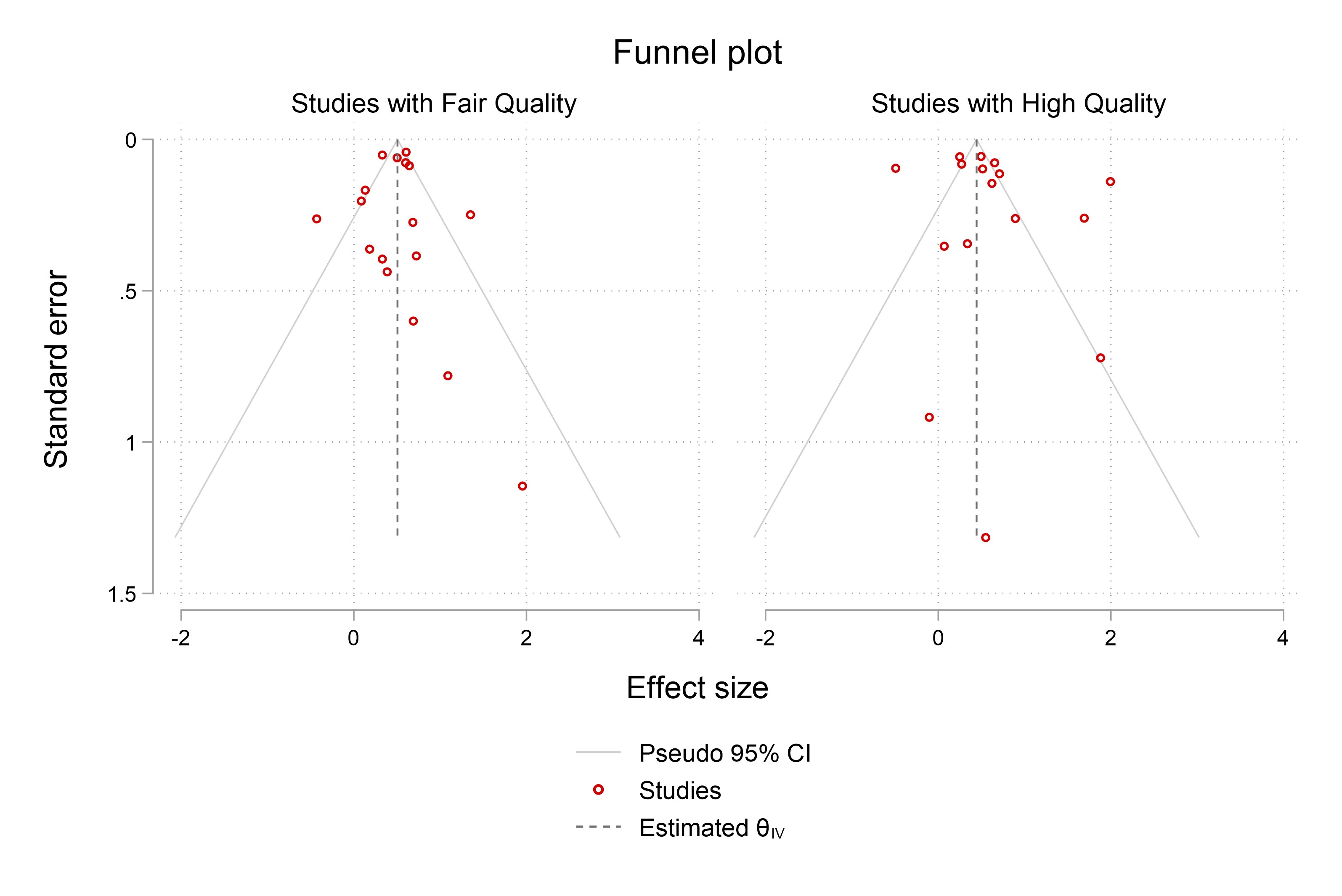

Supplement: Supplementary file 9 — Supplementary Material 9 Supplementary Fig. 2.d: Funnel plots for the Odds Ratio by quality of the study [file 737_2026_1739_MOESM9_ESM.jpg]
