# Supplementary material for: Prenatal exposure to nicotine and postpartum depression: a systematic review and meta-analysis
Source: Arch Womens Ment Health. 2026 Jul 1;29(4):102. doi: 10.1007/s00737-026-01739-6 (PMC13323114; doi:10.1007/s00737-026-01739-6)
Supplement: Supplementary file 10 — Supplementary Material 10 [file 737_2026_1739_MOESM10_ESM.docx]

Supplementary Table 1: Type of Exposure to Nicotine included in this systematic review and meta-analysis

| **Exposure** | **Number of Studies** |
| --- | --- |
| Active tobacco smoking | 22 |
| Secondhand smoking | 1 |
| Active tobacco smoking & Secondhand smoking | 4 |
| Electronic Nicotine Products use | 1 |
| Nicotine Dependence | 1 |
| Total | 29 |
