# Supplementary material for: Prenatal exposure to nicotine and postpartum depression: a systematic review and meta-analysis
Source: Arch Womens Ment Health. 2026 Jul 1;29(4):102. doi: 10.1007/s00737-026-01739-6 (PMC13323114; doi:10.1007/s00737-026-01739-6)
Supplement: Supplementary file 11 — Supplementary Material 11 [file 737_2026_1739_MOESM11_ESM.docx]

Supplementary Table 2: Meta-analysis pooled estimates of prevalence by subgroup analysis

|  | Exposed group | | Control/Comparison group | | |
| --- | --- | --- | --- | --- | --- |
| Subgroup | Pooled Prevalence | 95%CI | | Pooled Prevalence | 95%CI |
| **All studies** | 0.23 | [0.19 - 0.27] | | 0.14 | [0.13 - 0.17] |
| **Type of Exposure** |  |  | |  |  |
| Studies assessed for ATS | 0.25 | [0.19 – 0.31] | | 0.13 | [0.11 – 0.15] |
| Studies assessed for SHS | 0.10 | [0.09 – 0.11] | | 0.09 | [0.08 – 0.09] |
| Studies assessed for ENP (one study) |  |  | |  |  |
| **Method of assessment** |  |  | |  |  |
| Studies used PRAMS tool | 0.17 | [0.11 – 0.24] | | 0.11 | [0.09 – 0.13] |
| Studies used EPDS | 0.28 | [0.19 – 0.39] | | 0.17 | [0.12 – 0.22] |
| PHQ9 | 0.34 | [0.29 – 0.40] | | 0.13 | [0.11 – 0.16] |
| Diagnosis (one study) |  |  | |  |  |
| **Study design** |  |  | |  |  |
| Cross-sectional | 0.31 | [0.22 – 0.40] | | 0.16 | [0.13 – 0.19] |
| Cohort | 0.15 | [0.10 – 0.20] | | 0.12 | [0.09 – 0.16] |
| Case-control | 0.21 | [0.20 – 0.22] | | 0.13 | [0.12 – 0.13] |
| **Study Site** |  |  | |  |  |
| United States | 0.17 | [0.14 – 0.21] | | 0.13 | [0.11 – 0.15] |
| Asia | 0.21 | [0.11 – 0.34] | | 0.09 | [0.05 – 0.14] |
| South America | 0.32 | [0.21 – 0.44] | | 0.26 | [0.21 – 0.32] |
| Africa (one study) |  |  | |  |  |
| Europe (one study) |  |  | |  |  |
