# Supplementary material for: Prenatal exposure to nicotine and postpartum depression: a systematic review and meta-analysis
Source: Arch Womens Ment Health. 2026 Jul 1;29(4):102. doi: 10.1007/s00737-026-01739-6 (PMC13323114; doi:10.1007/s00737-026-01739-6)
Supplement: Supplementary file 12 — Supplementary Material 12 [file 737_2026_1739_MOESM12_ESM.docx]

Supplementary Table ‎3: Meta-analysis pooled estimates of the Odds Ratio by subgroup analysis

| Study | Exp (log OR): Estimated OR | 95%  Confidence Interval | P-value |
| --- | --- | --- | --- |
| Overall | 1.74 | [1.43 - 2.12] |  |
| **Type of Nicotine use** |  |  |  |
| Studies assessed for ATS | 1.96 | [1.59 – 2.41] | <0.001 |
| Studies assessed for SHS | 1.22 | [0.69 – 2.18] | 0.490 |
| Studies assessed for ENP (one study) | 1.14 | [0.82 – 1.58] | 0.430 |
| **Method of PPD ascertainment** |  |  |  |
| Studies used EPDS | 2.01 | [1.36 – 2.97] | <0.001 |
| Studies used PRAMS tool | 1.64 | [1.47 – 1.83] | <0.001 |
| Other methods | 1.66 | [1.01 – 2.72] | <0.001 |
| **Study Site** |  |  |  |
| US | 1.67 | [1.49 – 1.87] | <0.001 |
| Non-US | 2.26 | [1.47 – 3.48] | <0.001 |
| **Study design** |  |  |  |
| Cross-sectional/Case-control | 1.92 | [1.53 – 2.42] | <0.001 |
| Cohort | 1.34 | [0.97 – 1.86] | =0.07 |
| **Study Quality** |  |  |  |
| High Quality | 1.89 | [1.35 – 2.65] | <0.001 |
| Fair Quality | 1.60 | [1.33 – 1.93] | <0.001 |
