# Supplementary material for: Prenatal exposure to nicotine and postpartum depression: a systematic review and meta-analysis
Source: Arch Womens Ment Health. 2026 Jul 1;29(4):102. doi: 10.1007/s00737-026-01739-6 (PMC13323114; doi:10.1007/s00737-026-01739-6)
Supplement: Supplementary file 13 — Supplementary Material 13 [file 737_2026_1739_MOESM13_ESM.docx]

Supplementary Table 4: Leave-One-Out sensitivity analysis

| **Omitted study** | **OR** | **95% Confidence Interval** | **P-value** |
| --- | --- | --- | --- |
| Study 1 | 1.74 | [1.42 - 2.13] | 0.000 |
| Study 2 | 1.76 | [1.44 - 2.16] | 0.000 |
| Study 3 | 1.75 | [1.43 - 2.14] | 0.000 |
| Study 4 | 1.71 | [1.41 - 2.08] | 0.000 |
| Study 5 | 1.73 | [1.42 - 2.11] | 0.000 |
| Study 6 | 1.63 | [1.39 - 1.92] | 0.000 |
| Study 7 | 1.74 | [1.43 - 2.12] | 0.000 |
| Study 8 | 1.68 | [1.39 - 2.02] | 0.000 |
| Study 9 | 1.73 | [1.41 - 2.11] | 0.000 |
| Study 10 | 1.74 | [1.42 - 2.13] | 0.000 |
| Study 11 | 1.80 | [1.49 - 2.18] | 0.000 |
| Study 12 | 1.74 | [1.42 - 2.13] | 0.000 |
| Study 13 | 1.82 | [1.51 - 2.18] | 0.000 |
| Study 14 | 1.73 | [1.42 - 2.10] | 0.000 |
| Study 15 | 1.75 | [1.44 - 2.14] | 0.000 |
| Study 16 | 1.77 | [1.44 - 2.16] | 0.000 |
| Study 17 | 1.77 | [1.45 - 2.16] | 0.000 |
| Study 18 | 1.74 | [1.42 - 2.12] | 0.000 |
| Study 19 | 1.74 | [1.42 - 2.12] | 0.000 |
| Study 20 | 1.76 | [1.44 - 2.15] | 0.000 |
| Study 21 | 1.75 | [1.44 - 2.14] | 0.000 |
| Study 22 | 1.73 | [1.42 - 2.12] | 0.000 |
| Study 23 | 1.75 | [1.43 - 2.14] | 0.000 |
| Study 24 | 1.74 | [1.42 - 2.13] | 0.000 |
| Study 25 | 1.77 | [1.45 - 2.16] | 0.000 |
| Study 26 | 1.75 | [1.43 - 2.14] | 0.000 |
| Study 27 | 1.76 | [1.44 - 2.14] | 0.000 |
| Study 28 | 1.75 | [1.43 - 2.14] | 0.000 |
| Study 29 | 1.74 | [1.43 - 2.12] | 0.000 |
| Study 30 | 1.77 | [1.44 - 2.16] | 0.000 |
| Study 31 | 1.74 | [1.42 - 2.13] | 0.000 |
| Study 32 | 1.70 | [1.40 - 2.06] | 0.000 |
| Study 33 | 1.77 | [1.45 - 2.17] | 0.000 |
|  |  |  |  |
| exp(theta) | 1.74 | [1.43 - 2.12] | 0.000 |
