# Supplementary material for: Prenatal exposure to nicotine and postpartum depression: a systematic review and meta-analysis
Source: Arch Womens Ment Health. 2026 Jul 1;29(4):102. doi: 10.1007/s00737-026-01739-6 (PMC13323114; doi:10.1007/s00737-026-01739-6)
Supplement: Supplementary file 14 — Supplementary Material 14 [file 737_2026_1739_MOESM14_ESM.docx]

Supplementary Table ‎5: Show the estimated Odds Ratio after correction for potential publication bias using Trim and Fill Analysis

| **Studies** | **exp(ES): OR** | **95% Confidence Interval** |
| --- | --- | --- |
| Observed | 1.61 | [1.56 - 1.67] |
| Observed and imputed | 1.57 | [1.52 - 1.63] |
